# Screening for prostate cancer: protocol for updating multiple systematic reviews to inform a Canadian Task Force on Preventive Health Care guideline update

**DOI:** 10.1186/s13643-022-02099-9

**Published:** 2022-10-26

**Authors:** Alexandria Bennett, Andrew Beck, Nicole Shaver, Roland Grad, Allana LeBlanc, Heather Limburg, Casey Gray, Ahmed Abou-Setta, Scott Klarenbach, Navindra Persaud, Guylène Thériault, Brett D. Thombs, Keith J. Todd, Neil Bell, Philipp Dahm, Andrew Loblaw, Lisa Del Giudice, Xiaomei Yao, Becky Skidmore, Elizabeth Rolland-Harris, Melissa Brouwers, Julian Little, David Moher

**Affiliations:** 1 School of Epidemiology and Public Health, Faculty of Medicine, University of Ottawa, Ottawa, Ontario Canada; 2 Department of Family Medicine, McGill University, Montreal, Quebec Canada; 3 Global Health and Guidelines Division, Public Health Agency of Canada, Ottawa, Canada; 4 Department of Community Health Sciences, Max Rady College of Medicine, University of Manitoba, Winnipeg, Manitoba Canada; 5 Department of Medicine and Dentistry, University of Alberta, Edmonton, Alberta Canada; 6 Department of Family and Community Medicine, St. Michael’s Hospital, Toronto, Ontario Canada; 7 Lady Davis Institute of the Jewish General Hospital and Faculty of Medicine, McGill University, Montreal, Quebec Canada; 8 Department of Family Medicine, University of Alberta, Edmonton, Alberta Canada; 9 Urology Section, Minneapolis VA Healthcare System and Department of Urology, University of Minnesota, Minneapolis, Minnesota USA; 10 Evaluative Clinical Sciences, Odette Cancer Research Program, Sunnybrook Research Institute, University of Toronto, Toronto, Ontario Canada; 11 Department of Family and Community Medicine, Sunnybrook Health Sciences Centre, University of Toronto, Toronto, Ontario Canada; 12 Department of Health Research Methods, Evidence, and Impact, McMaster University, Hamilton, Ontario Canada; 13 Independent Information Specialist, Ottawa, Ontario Canada; 14 Clinical Epidemiology Program, Ottawa Hospital Research Institute, Ottawa, Ontario Canada

**Keywords:** Systematic review, Adults, Guideline, Primary care, Prostate cancer, Screening, Prostate-specific antigen

## Abstract

**Purpose:**

To inform updated recommendations by the Canadian Task Force on Preventive Health Care on screening for prostate cancer in adults aged 18 years and older in primary care. This protocol outlines the planned scope and methods for a series of systematic reviews.

**Methods:**

Updates of two systematic reviews and a de novo review will be conducted to synthesize the evidence on the benefits and harms of screening for prostate cancer with a prostate-specific antigen (PSA) and/or digital rectal examination (DRE) (with or without additional information) and patient values and preferences. Outcomes for the benefits of screening include reduced prostate cancer mortality, all-cause mortality, and incidence of metastatic prostate cancer. Outcomes for the harms of screening include false-positive screening tests, overdiagnosis, complications due to biopsy, and complications of treatment including incontinence (urinary or bowel), and erectile dysfunction. The quality of life or functioning (overall and disease-specific) and psychological effects outcomes are considered as a possible benefit or harm. Outcomes for the values and preferences review include quantitative or qualitative information regarding the choice to screen or intention to undergo screening. For the reviews on benefits or harms, we will search for randomized controlled trials, quasi-randomized, and controlled studies in MEDLINE, Embase, and the Cochrane Central Register of Controlled Trials. For the review on values and preferences, we will search for experimental or observational studies in MEDLINE, Embase, and PsycInfo. For all reviews, we will also search websites of relevant organizations, gray literature, and reference lists of included studies. Title and abstract screening, full-text review, data extraction, and risk of bias assessments will be completed independently by pairs of reviewers with any disagreements resolved by consensus or by consulting with a third reviewer. The GRADE (Grading of Recommendations Assessment, Development and Evaluation) approach will be used to assess the certainty of the evidence for each outcome.

**Discussion:**

The series of systematic reviews will be used by the Canadian Task Force on Preventive Health Care to update their 2014 guideline on screening for prostate cancer in adults aged 18 years and older.

Systematic review registration

This review has been registered with PROSPERO (CRD42022314407) and is available on the Open Science Framework (osf.io/dm32k).

**Supplementary Information:**

The online version contains supplementary material available at 10.1186/s13643-022-02099-9.

## Purpose and background

The Canadian Task Force on Preventive Health Care (“Task Force”) reviews and considers updating previous guideline recommendations periodically or if they identify important new evidence. The purpose of this evidence synthesis protocol is to plan a series of systematic reviews on prostate cancer screening for adults aged 18 years and older to inform an update to the 2014 Task Force Recommendations on screening for prostate cancer with the prostate-specific antigen (PSA) test [1].

### Description of the condition

The prostate is a small gland that is located just below the bladder and in front of the rectum. As a part of the male reproductive system, the function of the prostate is to secrete fluid to help form semen [2]. Changes to the prostate cells can cause cancerous, non-cancerous or benign conditions (e.g., benign prostatic hyperplasia), or pre-cancerous conditions (e.g., atypical small acinar proliferation). Adenocarcinoma of the prostate is the most common non-skin cancer in men in Canada and accounts for 95% of all prostate cancers [2, 3]. Rare cancerous or malignant tumors of the prostate can also develop (e.g., urothelial carcinoma).

Staging prostate cancer helps determine treatment and prognosis. This is based on the size of the tumor, lymph node involvement, and presence of distant metastases. The most commonly used system is the tumor, node, and metastasis staging system [2–4]. Staging can also be described as localized (Stage 1: T1 or T2a, N0, and M0 or Stage 2: T2b or T2c, N0, and M0), locally advanced (Stage 3: T3, N0, and M0 or Stage 4: T4, N0, and M0 or any T, N1, and M0), or metastatic (Stage 4; any T, any N, M1a, M1b, or M1c). Most prostate cancers (75%) are localized (Stage I or Stage II) at diagnosis in Canada [5, 6]. Besides staging, other prognostic information comes from grading. Grading prostate cancer describes how the cancer cells appear and function compared with non-cancerous cells. There are 3 histological grades given to cancerous cells (grades 3 to 5) based on their differentiation (the more abnormal pattern gives a higher value). The Gleason score is derived from the addition of the grade scores given to two most common cell patterns observed on biopsy samples. If there are more than two patterns, the most common pattern is added to the highest pattern. A Gleason score of 6 (3+3) represents a Gleason grade group 1 cancer, a score of 7 (3+4) grade group 2 cancer, a score of 7 (4+3) grade group 3 cancer, a score of 8 (4+4) grade group 4 cancer, and a score of ≥9 grade group 5 cancer [2, 7]. A higher Gleason grade group represents a more aggressive tumor. Although the original Gleason grading system is still widely referenced in the literature, there has been a consensus to adopt the International Society of Urological Pathology (ISUP) grading system which is represented by grade groups 1 to 5 [7].

### Disease burden and prevalence

Prostate cancer is the third highest cause of cancer-related mortality in individuals with a prostate [5, 8]. Globally, prostate cancer mortality rates are decreasing in high-income countries; however, the measured incidence and burden of disease (i.e., years of life lost, years lived with disability, and disability-adjusted life years) are increasing [9]. In Canada, approximately 1 in 9 individuals with prostate are expected to be diagnosed with prostate cancer in their lifetime and 1 in 29 will die from the disease [5]. The predicted 5-year estimated net survival for prostate cancer is 93% (95% CI, 92–93%), while the 10-year estimated net survival is 90% (95% CI, 89–90%) from 2012 to 2014 [5].

The measured incidence and mortality related to prostate cancer increase with age, and almost all (99%) prostate cancers are expected to occur among those who are 50 years of age or older [5]. Autopsy studies suggest that older adults have undiagnosed prostate cancer at the time of death, indicating that not all prostate cancers become clinically important [10]. From 2001 to 2011, the age-standardized incidence rate decreased by 1.6% per year, and then from 2011 to 2015, the age-standardized incidence rate declined more rapidly by 9.3% per year [5]. In 2016, there were 16,745 new cases of prostate cancer in Canada (excluding Quebec; age-standardized incidence rate of 120.9 per 100,000). Projection models estimate an age-standardized incidence rate of 116.7 per 100,000 in 2020 (excluding Quebec), which represents 23,300 new cases and 20% of all new potential cancer cases in men [5, 8, 11]. Age-standardized mortality rates have also declined over time in Canada, with 1.7% decrease per year between 1992 and 2001, and a more rapid 2.9% decline from 2001 to 2015 [6]. The cause of a reduction in mortality is unknown; however, it could be due to a decreased incidence, improved screening, treatment, or socioeconomic/environmental factors [5, 12]. From 2005 to 2015, the age-standardized incidence rate of stage I and stage II cancers decreased in Alberta and Manitoba, Canada, by an average of 3.2% per year, while the age-standardized incidence rate of later-stage prostate cancers (i.e., stage III and stage IV) remained stable [6]. Similar trends were seen in the USA with a decreased incidence of localized cancers and stable incidence of stage III disease [13]. However, retrospective data shows that the incidence of stage IV prostate cancer was observed to increase from 29 to 37 cases per 100,000 in the USA between 2010 and 2016 [13].

#### Natural history and risk factors

Despite the significant burden of prostate cancer, questions remain regarding its etiology. The disease is heterogenous in its clinical behavior, with disease progression dependent on tumor stage, tumor grade, and serum PSA concentration [14]. Data from the pre-screening era show that most prostate cancers are of low grade and grow slowly in the first 10–15 years following diagnosis and are not thought to metastasize, generating an appeal for active surveillance [14]. However, cancers with high-grade scores tend to progress more rapidly, and the 5-year relative survival for late-stage cancers that have metastasized is 28% [2]. The slow-growing nature of many tumors presents opportunities for clinical intervention through treatment or indicates that treatment may not be necessary.

There are a few well-known risk factors for the disease [15]. A family history of the disease is associated with an increased risk of prostate cancer, with an estimated 20% of cases reporting familial prostate cancer [15, 16]. While this suggests a heritable component, shared environmental risk factors also contribute to this risk [15, 17]. Genome-wide association studies have found several single nucleotide polymorphisms at genetic loci associated with an increased risk of the disease [15, 17, 18]. Individuals with germline mutations in the breast cancer predisposition genes, BRCA2 or BRCA1, have approximately a 20% and 9.5% lifetime risk of developing prostate cancer compared to non-carriers of the mutation, respectively [17]. Individuals may be more susceptible to prostate cancer based on their race and ethnicity. The prevalence of prostate cancer varies widely between racial and ethnic groups, with the highest prevalence among Black Americans with 185 cases per 100,000 compared to 107 cases per 100,000 in White Americans [15, 16, 18, 19]. In an American multiple-cohort study, results suggested that Black adults are at an absolute increased risk of prostate cancer-specific mortality of 0.5% (95% CI, 0.2–0.9%) compared with White men at 10 years post-diagnosis [20]. This increase in risk may be partially attributed to associated socioeconomic barriers to quality care [15, 16, 18, 20].

There is a lack of consistency in the body of evidence for other potential prostate cancer risk factors. Mixed evidence exists for lifestyle-related risk factors such as alcohol [15], tobacco smoking [21, 22], and dietary factors, including high saturated fat intake [15, 23] and micronutrient deficiency [15, 16]. The 2015 Canadian ComPARe study found that excess weight was a probable risk factor for prostate cancer [24]. Several industrial and occupational exposures have been suggested to cause an increased risk of prostate cancer, including pesticides, chromium, and shift work [23, 25]. There is also conflicting evidence that benign prostate hyperplasia (BPH) is a risk factor for prostate cancer [26–29]. Some studies suggest an association between BPH and risk for prostate cancer; however, it still remains unclear whether BPH is considered a risk factor [30]. Finally, some studies suggest an association between vasectomy and increased risk, but a causal relationship is not proven [15, 16, 23].

### Approaches to screening

#### PSA tests

The PSA test is a blood test that was first introduced to clinical practice to aid in the diagnosis and management of prostate cancer in 1986 and by 1990 it was used in Canada and the USA for prostate cancer screening [31, 32]. In 1991, a non-randomized study advocated screening for prostate cancer [33], and in the following year, the American Cancer Society formally supported the use of the PSA test for this purpose [34]. By 1994, the PSA test was approved for prostate cancer screening by the U.S. Food and Drug Agency [35]. PSA screening for prostate cancer became widely accepted, but there remain uncertainties and conflicting guidance surrounding its benefits and harms [36, 37].

The most common way to screen for prostate cancer is by measuring the total PSA concentration in the blood. PSA is a serine protease enzyme secreted by the epithelial cells in the prostate gland and is either bound to other proteins or free (unbound) within the blood [38]. Some guidelines propose thresholds for PSA levels in a screening context by age and race-specific cutoff values for further testing; however, no uniform cutoff value for PSA can be recommended for all men [39, 40]. Diagnoses other than cancer can explain false positive elevations in PSA and should be considered. These include a benignly enlarged prostate, urinary tract infection, or a recent digital rectal exam [38, 41, 42]. Although PSA has been widely used to screen for prostate cancer, there have been some challenges with PSA screening. Many studies have evaluated a one-point-in-time PSA value for screening or risk assessment to make decisions regarding prostate biopsy; however, PSA can be repeated and considered in conjunction with other factors such as symptoms or prostate size. One study noted that 25% of patients with an abnormal PSA have a normal PSA when the text is repeated [43].

#### Digital rectal examinations (DRE)

The DRE is a physical examination often used alongside the PSA test as a primary screening method in clinical practice [44]. Prior to PSA screening, DRE was the fundamental method of prostate cancer screening; however, by the 1990s, both PSA and DRE were used in conjunction for screening as DRE alone was deemed ineffective to detect prostate cancer [45]. A Canadian survey noted considerable differences in teaching methods for performing DRE, suggesting that using DRE alone as a screening tool is not possible due to inadequate physician training [46].

#### Alternative strategies

Using other biomarkers to determine an individual’s risk of developing prostate cancer has been a relatively new approach and has not yet been applied in general population screening; however, many RNA, DNA, and protein-based biomarkers in the urine, blood, and tissue samples have been studied to help predict the risk of prostate cancer [47–57]. Other tools or approaches for screening (coupled with PSA) for identifying adults at elevated risk include multiparametric magnetic resonance imaging (mpMRI), PSA velocity (PSAV), or risk calculators such as the Stockholm-3 model (S3M or STHLM3) [58–61]. PSA density, the PSA value (in ng/ml) divided by prostate volume (in CC), is another strategy used to predict prostate cancer, but has not been consistent in daily clinical practice over the years [62]. The use of PSA density has shown to add to the diagnostic value of clinically significant prostate cancer [62, 63].

Graphical prostate cancer risk calculators are prediction tools that have been developed to help physicians identify individuals at high risk of developing life-threatening prostate cancer, and who may benefit from early detection and/or treatment. Identification of individuals placed at high risk using a risk calculator may include risk factors for prostate cancer (e.g., family history) or via testing of various biomarkers [64]. In a systematic review to inform the 2018 U.S. Preventive Services Task Force (USPSTF) guidelines, the use of risk calculators in combination with PSA-based screening compared with PSA-based screening alone was evaluated to determine whether either screening method accurately identifies adults with clinically important tumors [37]. The systematic review included studies that used the Prostate Cancer Prevention Trial (PCPT) risk calculator or the European Randomized Study of Screening for Prostate Cancer (ERSPC) risk calculator [65, 66]. Both calculators were better at discriminating between patients with and without cancer than PSA alone; however, the accuracy of predicted risk probabilities either overestimated or underestimated actual risk in several cohorts for each calculator and have not been adjusted for key populations such as by race or ethnicity [37]. Studies have evaluated the validity of using these risk calculators; however, it’s important to note that none of these studies were done in the context of a screening trial [67, 68].

#### Benefits and harms of screening

Screening would be beneficial if it led to a successful detection of prostate cancer at an earlier, potentially more treatable, stage, and if it reduced morbidity and mortality associated with cancer in individuals who perceive themselves as healthy. Results from a large European randomized controlled trial found that PSA as a primary screening test in men aged 55 to 69 years of age (followed by prostate biopsies for adults with elevated PSA) significantly reduced prostate cancer mortality (rate ratio = 0.80 (95% CI, 0.72–0.89). The cumulative mortality rate was estimated to be 4.4 deaths per 10,000 person-years in the screening group compared to 5.5 deaths per 10,000 in the control group at 13 years follow-up and 5.3 deaths per 10,000 person-years in the screening group compared to 6.6 deaths per 10,000 person-years in the control group at 16 years follow-up [69]. In comparison, another large American randomized controlled trial found that PSA and DRE screening in men aged 55 to 74 years of age did not reduce prostate cancer mortality after 13 years of follow-up [70]. The study found cumulative mortality rates of 3.7 deaths per 10,000 person-years in the screening group compared to 3.4 deaths per 10,000 person-years in the usual care group (relative risk = 1.09, 95% CI, 0.87–1.36) [70].

Although screening for prostate cancer may detect high-risk cancer at an earlier stage, the benefits of prostate cancer screening remain unclear as the evidence suggesting decreased prostate cancer mortality in the literature is conflicting and potential gains in life expectancy are met with potential losses in quality of life [71]. A systematic review and meta-analysis found that PSA-based screening in men without a diagnosis of prostate cancer led to prostate-specific mortality in 2 of 1000 men in the screening group and 3 of 1000 men in the no screening group over 10 years (incidence rate ratio= 0.79, 95% CI 0.69–0.91, moderate quality evidence) [72]. The benefits of screening should be weighed against the potential harms. These include the possibility of detecting prostate cancers that would not have caused health concerns, known as overdiagnosis [71, 73, 74]. Overdiagnosis of prostate cancer may lead to overtreatment of indolent cancers (low-risk prostate cancers that would have never given rise to symptoms) [71, 75]. Certain treatments for prostate cancer, such as radical prostatectomy and radiotherapy, have various degrees of acute and long-term side effects including urinary incontinence, erectile dysfunction/impotence, and bowel disturbance, all of which have the potential to negatively impact the quality of life [76]. Due to the low probability of the PSA test correctly identifying men who do not have prostate cancer (specificity), many men may be falsely identified (false positives) as possibly having prostate cancer and then subjected to unnecessary biopsies [77]. Differences in the accuracy of the PSA test are partially due to the PSA threshold used to signal a need for further testing for prostate cancer, as some thresholds may increase the detection of cancers that are unlikely to cause health concerns even if left untreated [78]. The American Cancer Society completed a series of systematic reviews and found that PSA screening for any prostate cancer with a cutoff of 4.0 ng/mL resulted in 21% of screening tests that identified prostate cancer being truly positive (sensitivity) and 91% of screening tests that did not identify prostate cancer being truly negative (specificity) [79]. Lowering the PSA cutoff to 3.0 ng/mL increased sensitivity to 32%, but worsened specificity to 85%, resulting in an increase of false positive cases [79]. Although there is variability in the diagnostic accuracy of the PSA test, the specificity of PSA testing for detecting clinically important prostate cancer may be improved when followed by other assessment methods, such as a risk calculator [80] or mpMRI [81].

#### Evidence-based guideline recommendations

In 2014, the Canadian Task Force published recommendations on screening for prostate cancer in adults not previously diagnosed with prostate cancer. Based on systematic review evidence, the Task Force provided a recommendation against screening for prostate cancer using the PSA test for adults less than 55 years of age (strong recommendation; low-quality evidence), adults 55 to 69 years of age (weak recommendation; moderate quality evidence), and adults 70 years of age and older (strong recommendation; low-quality evidence). The Working Group concluded the evidence did not conclusively show that screening with the PSA test reduced prostate cancer mortality, whereas evidence suggested an increased risk of harm in individuals younger than 55 years of age or older than 70 years of age. For adults aged 55 to 69 years, the Task Force recommended against screening (weak recommendation; moderate quality evidence) because there was conflicting evidence that suggested a small potential benefit of screening in one study, while other studies found no benefit. The recommendations for individuals aged 55 to 69 placed a relatively low value on a small potential absolute decrease in prostate cancer mortality and reflected concerns with false-positive results, unnecessary biopsies, overdiagnosis of prostate cancer, and harms associated with unnecessary diagnostic tests and treatment [1]. Since the publication of the 2014 Task Force recommendations, there have been new recommendations from other organizations (see Table 1 and Additional File 1).

**Table 1:** Selected summary of evidence-based guideline recommendations

Organization	Year	Country	Recommendation
Canadian Task Force on Preventive Health Care	2014	Canada	<55 or ≥70: Recommended against screening (strong recommendation, low-quality evidence) 55 to 69: Recommended against screening (weak recommendation; moderate quality evidence)
American College of Physicians	2015	USA	<50 or ≥70: Recommended against screening 50 to 69: Recommended shared decision making
Members of the rapid recommendation panel	2018	International	Recommended against systematic PSA-based screening (weak recommendation). Shared decision making is needed for men considering screening.
U.S. Preventive Services Task Force	2018	USA	55 to 69: Recommended shared decision making ≥70: Recommended against screening
American Academy of Family Physicians	2019	USA	Recommended against screening. For individuals aged 55 to 69 and considering periodic screening: Recommended shared decision making
UK National Screening Committee	2020	UK	Recommended against systematic population screening

In 2015, the American College of Physicians (ACP) recommended against routine PSA screening for adults aged 50 to 69 years of age who have not had an informed discussion with their clinician and have not expressed a clear preference for testing [82]. The ACP also recommended against any testing for prostate cancer in adults aged less than 50 or greater than 69 years of age and adults of any age who are not in good health and have a life expectancy of less than 10 years [82]. In 2018, the USPSTF recommended that adults aged 55 to 69 years make an individual decision about whether to be screened for prostate cancer after discussing the potential benefits and harms with their clinician [40]. For adults 70 years of age and older, the USPSTF also recommended against routine screening because the potential benefits did not outweigh the expected harms [37, 40]. An international Rapid Recommendation panel also made a weak recommendation in 2018 against systematic PSA-based screening for prostate cancer and that shared decision making is needed for men considering the screening test [83]. In 2019, the American Academy of Family Physicians (AAFP) provided a recommendation against routine PSA-based screening for prostate cancer, a similar recommendation to the USPSTF. The AAFP noted that screening for PSA may prevent mortality in a small number of individuals, but it put many at risk for long-term harms, such as urinary incontinence and erectile dysfunction. For adults aged 55 to 69 years of age who are considering periodic screening, they recommended clinicians discuss the risks and benefits and then engage in shared decision-making [84]. In 2020, the UK NSC recommended against systematic population screening for prostate cancer of asymptomatic adults because the effectiveness of PSA screening on mortality was unclear and the PSA test may falsely identify individuals who do not have prostate cancer often leading to unnecessary tests, overdiagnosis, and/or treatment with harmful side effects [85].

#### Rationale, key questions, and approach

Since the release of the 2014 Task Force guideline for prostate cancer screening, a topic surveillance plan and scoping refinement exercise for the Working Group was led by the Science Team of the Global Health and Guidelines Division at the Public Health Agency of Canada (AL, HL, EH) to identify new guidelines and systematic reviews on PSA screening that have been published since 2014 (see Table 1 and Additional File 1). Based on this scoping and refinement exercise, the Task Force decided to update their 2014 guideline on prostate cancer screening since additional primary study evidence (e.g., the Cluster Randomized Trial of PSA Testing for Prostate Cancer) and additional follow-up from previously included studies (e.g., prostate, lung, colorectal, and ovarian cancer screening trial and ERSPC) have been published [69, 70, 86]. The scoping and topic refinement exercise informed the Task Force decision to use evidence from two recent systematic reviews with similar key questions (KQs) and eligibility criteria [87, 88] and to produce updated recommendations for primary care clinicians on screening for prostate cancer. The systematic review methodology for candidate reviews was reviewed and assessed, and necessary adjustments to the methodology were made to comply with the Task Force Methods [89]. For example, the UK NSC used one reviewer to screen citations followed by a second reviewer to validate included citations and 10% of the excluded citations. To ensure our review complies with Task Force methods, the UK NSC reviews will be enhanced by screening all previously included studies and those in the excluded studies list (see details in the “Methods” section).

**Fig. 1:**
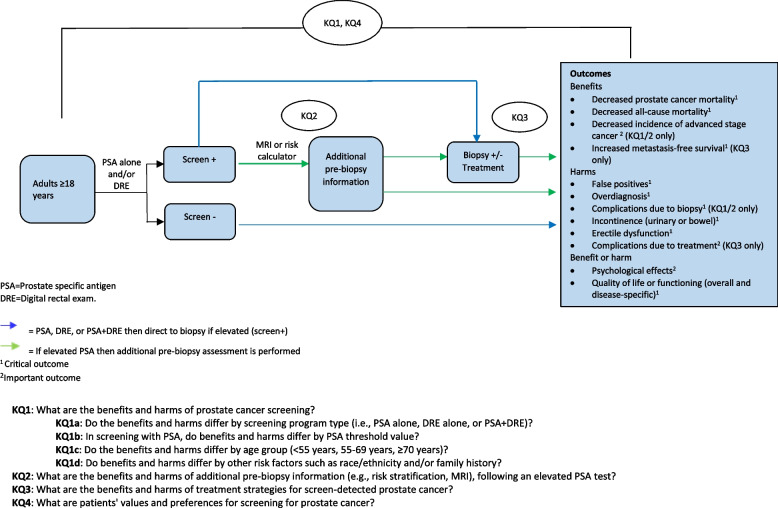
Analytical Framework – Screening for prostate cancer among adults ≥18 years

The proposed plan to update two systematic reviews and conduct a de novo review will address each of the KQ listed in Table 2 and will follow the PICOTS (population, interventions, comparators, outcomes, timing, and setting) criteria, which is further detailed in Tables 4, 5, 6, and 7 under the methods section. The analytic framework of the KQs, relevant population, interventions, and outcomes to be considered are also detailed in Fig. 1.

**Table 2:** Key questions to inform an update of recommendations by the Task Force on prostate cancer screening in adults aged 18 years and older

Key questions	
**KQ1**	What are the benefits and harms of prostate cancer screening? a) Do the benefits and harms differ by screening modalities (i.e., PSA alone, DRE alone, or PSA+DRE)? b) In screening with PSA, do benefits and harms differ by PSA threshold value? c) Do the benefits and harms differ by age group (<55 years, 55–69 years, ≥70 years)? d) Do the benefits and harms differ by other risk factors such as race/ethnicity and/or family history?
**KQ2**	What are the benefits and harms of incorporating additional information (e.g., risk stratification, MRI) into clinical decision making following an elevated PSA test?
**KQ3**	What are the benefits and harms of treatment strategies for screen-detected prostate cancer?
**KQ4**	What are patients’ values and preferences for screening for prostate cancer?

## Methods

The ERSC will complete the update of two systematic reviews as well as conduct one de novo review. For KQ1, the ERSC will conduct an update of the 2020 UK NSC review on prostate cancer screening with the integration of additional study designs and interventions from a 2021 Cochrane review [87]. For KQ2, the ERSC will conduct a de novo review. For KQ3, the Task Force Working Group will rely on the recent 2020 UK NSC review for prostate cancer screening [85]. For KQ4, we will conduct a review update of the 2018 systematic review conducted by Vernooij et al. [88].

### Protocol development

The methodology used for these reviews follows the Task Force methods’ manual [89], with guidance from the Cochrane Handbook [90] and GRADE Working Group [91]. Reporting of the protocol follows the Preferred Reporting Items for Systematic Reviews and Meta-Analyses Protocols (PRISMA-P) checklist [92]. The completed PRISMA-P checklist is included in Additional file 2. In addition, the protocol and relevant project materials are available on the Open Science Framework (osf.io/dm32k).

This protocol was developed by the Evidence Review and Synthesis Centre (ERSC) at the University of Ottawa (ABeck, ABennett, NS, DM, JL, MB) in consultation with the Prostate Cancer Screening Working Group consisting of Task Force members and fellows (AAS, RG, SK, NP, GT, BDT, KT), clinical experts (NB, PD, ALoblaw, LDG), and the Science Team of the Global Health and Guidelines Division at the Public Health Agency of Canada (ALeBlanc, HL, EH, CG).

Following the development of the scoping and refinement exercise led by the Science Team, the Working Group revised and finalized the key questions and related PICOTS with involvement from the entire Task Force, external clinical experts, the ERSC, and the Science Team (Fig. 1, Tables 2, 3, 4, 5, 6, and 7). The Working Group, external clinical experts, and Science Team will not be involved in the selection of studies, data extraction, or data analysis, but may be consulted (e.g., where input is needed for potentially eligible studies, we may consult those who are blinded to study identification information and outcome data). All final decisions will be made by the ERSC. The Canadian Task Force has approved the final version of this protocol and it has been reviewed by peer reviewers and stakeholders (see Additional file 7). Any amendments to the protocol will be provided in the final manuscript. The completed reviews will be reported using the PRISMA 2020 reporting guideline and appropriate PRISMA extensions (e.g., PRISMA-S) [93].

**Table 3:** Final set of outcomes deemed to be of critical or important for guideline development and decision-making

Outcomes^**a**^	Potential Benefit or Harm	Priority
**KQ1 & KQ2: What are the benefits and harms of prostate cancer screening?**		
Prostate cancer mortality	Benefit (decrease)	Critical
All-cause mortality	Benefit (decrease)	Critical
False positives	Harm	Critical
Overdiagnosis	Harm	Critical
Complications due to biopsy^b^	Harm	Critical
Quality of life or functioning (overall and disease-specific; using validated scales)^c^	Benefit (increase) or harm (decrease)	Critical
Incontinence (urinary or bowel)^c^	Harm	Critical
Erectile dysfunction^c^	Harm	Critical
Incidence of metastatic cancer^c^	Benefit (decrease)	Important
Psychological effects	Benefit or harm	Important
**KQ3: What are the benefits and harms of treatment strategies for screen-detected prostate cancer?**		
Prostate cancer mortality	Benefit (decrease)	Critical
All-cause mortality	Benefit (decrease)	Critical
Quality of life or functioning (overall and disease-specific; using validated scales)^c^	Benefit (increase) or harm (decrease)	Critical
Incontinence (urinary or bowel)^c^	Harm	Critical
Erectile dysfunction^c^	Harm	Critical
Rate of metastasis development	Benefit (decrease)	Important
Psychological effects	Benefit or harm	Important
Complications due to treatment (i.e., surgical adverse events, radiotherapy toxicity, endocrinological effects)	Harm	Important
**KQ4: What are patients’ values and preferences for screening for prostate cancer?**		
Values and preferences regarding the choice to screen, based on information overall benefits and harms (i.e., the benefit considered worthwhile to undergo burden/harm). Benefits must include mortality.	Does not apply	Not rated
Intention to undergo screening based on information regarding the patient-important outcomes presented in decision aids.	Does not apply	Not rated

^a^The following 2014 guideline outcomes are not included in the current guideline: overtreatment and false negatives

^b^“Complications due to biopsy” is a reworded version of the previous 2014 guideline outcome, “physical harms associated with screening (e.g., bleeding, infection, bruising)”

^c^These outcomes have been newly added since the previous 2014 guideline

**Table 4:** KQ1 eligibility criteria (benefits and harms of screening)

	Inclusion criteria	Exclusion criteria
**Population**	Individuals not known to be at elevated risk for prostate cancer. *Secondary analyses for decision-making*: • Screening interval (KQ1) • PSA thresholds (KQ1b) • Age: <55 years, 55-69 years, ≥70 years (KQ1c) • Race and/or ethnicity (KQ1d) • Obesity, as defined by study authors (KQ1d) • Family history (KQ1d)	Individuals <18 years. Individuals with a pre-existing or previous history of prostate cancer. Individuals specifically selected for the presence of another condition or risk factor (i.e., individuals working with chemicals known to be carcinogenic or individuals with known genetic markers). Individuals who have had a previous PSA screen and/or individuals with a “normal” change in urine function are not excluded. Normal will be defined by clinician judgment.
**Interventions**	One or more clinical or lab tests (e.g., PSA+DRE, PSA alone, DRE alone) with or without additional tests before the biopsy.	Other screening methods that do not include PSA or DRE.
**Comparator**	No screening Usual care Alternate type of screening within the options previously stated (e.g., DRE alone) [KQ1a]	N/A
**Outcomes**	Potential benefits 1. Reduced prostate cancer mortality 2. Reduced all-cause mortality 3. Reduced incidence of metastatic cancer Potential harms 4. False positives 5. Overdiagnosis^a^ 6. Complications due to biopsy 7. Incontinence (urinary or bowel) 8. Erectile dysfunction Either benefit or harm 9. Quality of life or functioning (overall and disease-specific*) 10. Psychological effects *As defined/reported by the study authors.* *scales with acceptable measurement properties (e.g., validity, reliability) for use in prostate cancer	N/A
**Timing of outcome assessment**	Any timing	N/A
**Setting**	Primary care settings	Settings not generalizable to primary care
**Study design**	Benefits and harms Randomized (including cluster RCTs), quasi-randomized, and controlled clinical trials Harms only Cohort studies (if needed)^b^	Editorials, commentaries, letters, conference proceedings, government reports, case series, case report, narrative reviews, systematic reviews^c^
**Language**	English or French	N/A
**Dates of publication**	All dates are included (as per the UK NSC search), however, the current update will include 2019 to present	N/A

^a^Outcome data for overdiagnosis will be extracted as reported by the study authors. When presenting results, overdiagnosis may be expressed as the number of over-diagnosed cancers over the following possible denominators: (1) the number of men screened, (2) the number of screen-detected cases, or (3) the number of prostate cancer cases

^b^If certainty in the evidence is a barrier to the development of recommendations and the CTFPHC believes that further evidence from cohort studies may influence their recommendations

^c^The reference list of relevant systematic reviews will be reviewed for relevant studies

**Table 5:** KQ2 eligibility criteria (benefits and harms of additional information following an elevated PSA test)

	Inclusion criteria	Exclusion criteria
**Population**	Individuals with an elevated* PSA test *definition of elevated to be determined by the included study *Secondary analyses for decision-making*: • PSA thresholds • Age: <55 years, 55–69 years, ≥70 years • Race and/or ethnicity • Obesity, as defined by the study authors • Family history	Individuals <18 years. Individuals with a history of prostate cancer. Individuals who have had a previous PSA screen are not excluded. Individuals specifically selected for the presence of another condition or risk factor (e.g., other types of cancer, individuals working with chemicals known to be carcinogenic, individuals with known genetic risk)
**Interventions**	Additional testing (e.g., risk stratification, MRI) Tests used alone, sequentially or in combination to determine the need for biopsy, including but not limited to clinical variables (e.g., age, family history of prostate cancer, a previous biopsy), ratio of free to total PSA, blood biomarkers (PSA, MIC1, etc.) or biomarker panels (4K panel, STHLM3 panel), urine biomarkers, genetic markers, DRE, prostate volume, imaging markers/techniques (e.g., mp-MRI), nomograms combining one or more of the above variables or tests.	Any post-biopsy intervention (e.g., MRI that stratifies risk of an already diagnosed cancer)
**Comparator**	No additional testing (PSA-based screening only (including single threshold PSA test, age-specific thresholds, variable screening intervals)) Usual care	N/A
**Outcomes**	Potential benefits 1. Reduced prostate cancer mortality 2. Reduced all-cause mortality 3. Reduced incidence of metastatic cancer Potential harms 4. False positives 5. Overdiagnosis^a^ 6. Complications due to biopsy 7. Incontinence (urinary or bowel) 8. Erectile dysfunction Either benefit or harm 9. Quality of life or functioning (overall and disease-specific*) 10. Psychological effects *As defined/reported by the study authors.* *Scales with acceptable measurement properties (e.g., validity, reliability) for use in prostate cancer	N/A
**Timing of outcome assessment**	Any timing	N/A
**Delivery setting**	Majority recruited from primary care settings	Settings not generalizable to primary care
**Study design**	RCTs (including cluster RCTs), observational studies with consecutively enrolled populations	Case reports, case series, systematic reviews^b^, narrative reviews, editorials, commentaries, letters, conference proceedings, government reports.
**Language**	English or French	N/A
**Dates of publication**	All dates	N/A

^a^Outcome data for overdiagnosis will be extracted as reported by study authors. When presenting results, overdiagnosis may be expressed as the number of over-diagnosed cancers over the following possible denominators: (1) the number of men screened, (2) the number of screen-detected cases, or (3) the number of prostate cancer cases

^b^The reference list of relevant systematic reviews will be reviewed for relevant studies

**Table 6:** KQ3 UK NSC 2020 eligibility criteria (benefits and harms of treatment)

	Inclusion criteria	Exclusion criteria
**Population**	Individuals over 18 years with localized (early stage) prostate cancer	N/A
**Interventions**	Any of the following interventions: surgery, high-intensity focused ultrasonography, radiation therapy, ablative therapy, hormonotherapy	Any non-approved or experimental treatment
**Comparator**	No treatment, the same intervention with a minor difference (e.g., difference in dose or scheduling), watchful waiting, or active surveillance	Any other comparators
**Outcomes**^**a**^	Potential benefits 1. Reduced prostate cancer mortality 2. Reduced all-cause mortality 3. Decreased rate of metastasis development Potential harms 4. Incontinence (urinary or bowel) 5. Erectile dysfunction 6. Complications due to treatment (i.e., surgical AEs, radiotherapy toxicity, endocrinological effects) Either benefit or harm 7. Quality of life or functioning (overall and disease-specific) 8. Psychological effects	N/A
**Timing of outcome assessment**	Not specified	N/A
**Delivery setting**	Any country	N/A
**Study design**	RCTs (including cluster RCTs), observational studies with consecutively enrolled populations	Case reports, case series, systematic reviews^b^, narrative reviews, editorials, commentaries, letters, conference proceedings, government reports.
**Language**	English	N/A
**Dates of publication**	Before 2019 (UK NSC)	Full-text studies published prior to 2018 or 2014 for the specific interventions were not included in the NICE systematic review.

^a^The UK NSC has indicated that the rate of disease recurrence after successful initial treatment is an outcome of interest; however, this outcome is not of interest to the CTFPHC

^b^The reference list of relevant systematic reviews will be reviewed for relevant studies

**Table 7:** KQ4 eligibility criteria (patient values and preferences of screening)

	Inclusion criteria	Exclusion criteria
**Population**	Individuals (>18 years of age) without a clinical suspicion of prostate cancer and without a history of prostate cancer.	Studies with a mixed population (individuals with prior history of screening and/or prior diagnosis) that do not separate information for our population of interest will be excluded.
**Exposure**	Experience with outcomes related to screening. Exposure to clinical scenarios or Information on PSA screening and/or screening attributes (e.g., presented in decision aids).	N/A
**Comparator**	Depending on the study design, a comparator may be: • No comparison. • Different types of clinical scenarios or information on screening.	N/A
**Outcomes**	Qualitative information about: • Values and preferences regarding the choice to screen, based on information overall benefits and harms (i.e., the benefit considered worthwhile to undergo burden/harm). Benefits must at least include prostate cancer mortality and/or incidence of cancer risk. • Intention to undergo screening based on information regarding the patient-important outcomes presented in decision aids.	N/A
**Timing**	Any timing.	N/A
**Setting**	Any setting.	N/A
**Study design**	Any experimental or observational study design (including abstracts if data is available) reporting patient preferences to screen or not to screen (e.g., discrete-choice experiments).	Studies reporting only outcome prioritization, time trade-off, health state values, or willingness to pay. Systematic reviews^a^, cost-effectiveness studies, qualitative studies, case report, and case series. Analyses of data that were not reported by patients (e.g., databases of health records) or on outcomes outside the perspective of individuals considering prostate cancer screening. Studies reporting only access to screening; studies on knowledge or awareness about screening.
**Language**	English or French	N/A
**Dates of publication**	All dates are included (as per the Vernooij et al. study); however, the current update will include 2017 to present.	N/A

^a^The reference list of relevant systematic reviews will be reviewed for relevant studies

### Outcome rating

Following the Task Force outcome rating process, members of the Working Group developed a list of preliminary outcomes of interest for KQ1, KQ2, and KQ3 starting with outcomes from the previous 2014 Task Force guideline [89]. Outcomes were rated by five Task Force Working Group members according to GRADE methodology as critical (rated 7 to 9 out of 9), important (rated 4 to 6 out of 9), or of limited importance (rated 1 to 3 out of 9) for making guideline recommendations [94]; then through a consensus process among the Task Force members, final rating levels were agreed upon. A summary of included outcomes and GRADE ratings are detailed in Table 3.

For KQ1 and KQ2, ten outcomes were selected as critical or important for guideline development and decision making (Table 3). This number of outcomes balanced considerations of GRADE methodology (i.e., limited capacity to consider multiple outcomes at once) [49] while including all patient-important outcomes. Based on ratings from the Working Group members, critical outcomes of interest for potential benefits of screening include reduced prostate cancer mortality and reduced all-cause mortality. Critical outcomes for the potential harms of screening include false positives, overdiagnosis, complications due to biopsy, incontinence (urinary or bowel), and erectile dysfunction. Quality of life or functioning (overall and disease-specific; using validated scales) is a critical outcome that will be assessed as either a potential benefit (observed increase) or potential harm (observed decrease). Outcomes rated as important include the reduced incidence of metastatic cancer as a potential benefit and psychological effects as either a potential benefit or harm.

For KQ3, eight outcomes were selected by the Working Group members using the same methods. Critical outcomes of interest for the benefits and harms of treatment strategies for screen-detected prostate cancer include reduced prostate cancer mortality and reduced all-cause mortality, while incontinence (urinary or bowel) and erectile dysfunction were considered potential harms. Quality of life or functioning (overall and disease-specific; using validated scales) is a critical outcome that will be measured as either a potential benefit (observed increase) or potential harm (observed decrease). Outcomes rated as important include decreased rate of metastasis development as a potential benefit, while complications due to treatment (i.e., surgical adverse events, radiotherapy toxicity, endocrinological adverse effects) are a harm. Psychological effects were rated as an **important** outcome and will be considered either a potential benefit or a potential harm. No outcomes were rated by the Working Group for KQ4, as we will rely on the outcomes of interest of the previous review by Vernooij et al. [88]. These include values and preferences regarding the choice to screen, overall benefits and harms, and intention to undergo screening based on information regarding patient-important outcomes presented in decision aids.

### Eligibility criteria

The inclusion and exclusion criteria for each KQ are described below in Tables 4, 5, 6, and 7. A review update will not be conducted for KQ3, and the Task Force will rely on the original eligibility criteria used by the UK NSC.

### Information sources and search strategy

The search strategies from selected SRs were modified and updated to reflect current standards. The new search strategies were developed and tested through an iterative process by an experienced medical information specialist (BS) in consultation with the ERSC review team. The searches will be performed from the previous reviews search dates, and the updated search strategies are available (Additional file 3) [87, 88]. The MEDLINE strategies for each KQ were peer-reviewed by another senior information specialist using the PRESS Checklist [95], and the completed PRESS checklist is in Additional file 4.

#### Key questions 1 and 2: What are the benefits and harms of prostate cancer screening?

For KQ1, we will update the 2020 UK NSC review on prostate cancer screening, with the integration of additional study designs (i.e., quasi-experimental cohort studies (harms only) and interventions (i.e., DRE alone) from a 2021 Cochrane review [87]. Searches for KQ1 were derived from the 2020 UK NSC review on prostate cancer screening and the 2013 Cochrane review by Ilic and colleagues [87]. For KQ2, we will conduct a de novo review. Using the multifile option on the OVID platform for both KQs, we will search Ovid MEDLINE® ALL, including Epub Ahead of Print, In-Process & Other Non-Indexed Citations, and Embase Classic+Embase, and EBM Reviews - Cochrane Central Register of Controlled Trials. The strategies will utilize a combination of controlled vocabulary (e.g., “Prostatic Neoplasms,” “Early Detection of Cancer,” “Digital Rectal Examination”) and keywords (e.g., “prostate cancer,” “screening,” “DRE”). We will apply the Cochrane Highly Sensitive Search Strategy for identifying randomized trials in MEDLINE, sensitivity- and precision-maximizing version (2008 revision), with slight amendments, to the Ovid strategies. We will also utilize a robust filter for non-randomized studies. Vocabulary and syntax will be adjusted across the databases, and animal-only, opinion pieces, and conference abstracts will be removed where applicable. At the time of execution, we will limit results to the date from the last searches to the present as appropriate for both the UK NSC and Cochrane reviews and the study designs in question. Results will be downloaded and deduplicated using EndNote X9.3.3 (Clarivate Analytics) [96]. Specific details regarding the draft search strategies are found in Additional file 3.

#### Key question 3: What are the benefits and harms of treatment strategies for screen-detected prostate cancer?

For KQ3, the ERSC will not undertake updated searches or additional syntheses and the Working Group will rely on the recent 2020 UK NSC review to answer KQ3 [87].

#### Key question 4: What are patients’ values and preferences for screening for prostate cancer?

For KQ4, we will update the 2018 systematic review by Vernooij and colleagues [88]. We will search for new evidence from the last search date of each review and adjust where required to meet the Task Force eligibility criteria.

Searches for KQ4 were derived from the review by Vernooij and colleagues [88]. Using the multifile option on the OVID platform, we will search Ovid MEDLINE® ALL, including Epub Ahead of Print, In-Process & Other Non-Indexed Citations, Embase Classic+Embase, and PsycInfo. The strategies utilize similar controlled vocabulary and keywords from KQ1 and KQ2 to describe the eligible population for prostate cancer screening. In addition, terminology pertaining to patient preferences and values will be incorporated. This includes controlled vocabulary (e.g., “Attitude to Health,” “Mass Screening/px [Psychology],” “Patient Preference”) and free-text terms (decision-making, empower, informed choice). We will remove animal-only records and conference abstracts where applicable and limit results from the date of the last Vernooij et al. searches to the present. Results will be downloaded and deduplicated using EndNote X9.3.3 (Clarivate Analytics) [96]. The search strategies are available in Additional file 3.

We will aim for literature saturation by supplementing the electronic database searches with gray literature sources and reviewing the references of included studies and relevant evidence-based clinical practice guidelines and systematic reviews identified during screening for additional records. We will search gray literature sources for unpublished documents using the Canadian Agency for Drugs and Technologies in Health (CADTH) Gray Matters checklist [97]. The checklist includes public health websites, health technology assessment agencies, clinical practice guideline organizations, clinical trial registries, search engines, and additional databases. In addition to the CADTH checklist, we will search websites of relevant organizations as suggested by the Working Group and clinical experts. The full list of relevant websites is available in Additional file 5.

### Study selection

For KQ1, KQ2, and KQ4, results from the literature searches will be uploaded to DistillerSR [98]. Following the development of the screening forms for study selection, we will pilot test the title and abstract screening and full-text article review forms on a random sample of 50 titles and abstracts and 25 full-text articles until reviewer agreement is high (>95%). Any discrepancies among reviewers will be resolved by discussion or consulting with a third reviewer and adjustments to the form will be completed as needed.

Title and abstract screening and full-text review will be completed independently and in duplicate by reviewers using the study eligibility screening forms. Any discrepancies will be resolved by consensus among the reviewers or by a third reviewer. For KQ1, to address limitations of the UK NSC rapid review methodology, previously included studies and studies excluded during full-text screening will also be screened independently to determine if they meet our inclusion criteria (e.g., previously excluded French language publications) [87, 88].

We will request articles that are not available electronically through the university library interlibrary loan service. Reports, where only abstract information is available, will be included if sufficient information is provided (i.e., outcomes). We will contact the corresponding author (by email every 2 weeks for a maximum of three attempts) of relevant conference abstracts and protocols for potential includable manuscripts or unpublished data. If a potentially relevant study reports information that is unclear for us to decide on eligibility, the corresponding author will be contacted for additional information (by email every 2 weeks for a maximum of three attempts). If no response is received, the article will be excluded. If advice is required on potentially eligible studies, we may consult with the Working Group and clinical experts on the study design and outcomes collected. When consulting with the Task Force Working Group, we will anonymize the article to avoid study identification and outcome data and the ERSC will decide on the final eligibility of these studies. For the quality of life and functioning outcomes, we will limit measurement instruments with acceptable measurement properties (e.g., face validity) or recommended instruments as advised in the literature. If multiple instruments are used, we will consult with the Working Group (blinded to the results) on the relevance of the available instruments and have them rate on a visual analog scale. The Working Group will make a priori determinations on the clinical utility of each disease-specific scale within a primary care setting. The Working Group will also help to create groupings for each validated scale, which will assist in grading the certainty of evidence.

For excluded studies during the full-text review, the reasons for exclusion will be agreed upon by reviewers and a list of excluded studies with reasons will be generated. We will document the selection process in a PRISMA flow diagram [99].

### Data extraction

For KQ1, KQ2, and KQ4, we will develop standardized extraction forms in DistillerSR [98]. Next, we will pilot-test the forms on a random sample of five included studies for each KQ. Any discrepancies among reviewers will be resolved by discussion or by consulting with a third reviewer and adjustments to the forms will be completed as needed.

For extracting data from newly included studies, the process will involve reviewers independently and in duplicate using the standardized extraction forms. Any differences will be resolved by consensus among the reviewers or by a third reviewer if consensus cannot be reached. We will rely on the primary study data reported in the previous reviews when possible. If not (e.g., missing outcome of interest), we will follow the extraction process described above for newly included studies (i.e., two reviewers with consensus). When extracting data from the previous reviews, one reviewer will extract the data, while another reviewer verifies the information (not including results data, which will be extracted independently by two reviewers with consensus). Information on the preliminary data items for each KQ (newly included studies only) is available in Additional File 6. Data will be re-formatted and presented in the text and tables of the final manuscript, as appropriate. Where information is missing or unclear, we will contact the authors of the study or previous reviews for additional information every two weeks for a maximum of three attempts. If there are multiple publications of the same study, we will extract data from the most recent publication and older publications will be used as secondary sources.

### Risk of bias assessment

For KQ1, KQ2, and KQ4, we will create quality assessment extraction forms in DistillerSR [98]. Reviewers will pilot each form on a random sample of five included studies for each KQ. Any conflicts will be resolved by discussion.

For de novo assessments, two reviewers will independently appraise the risk of bias using the matching tool from the relevant previous reviews for the study design of the included studies (e.g., version 2 of the Cochrane Risk of Bias tool for randomized trials). Any disagreements in the assessments will be resolved by consensus or by consulting with a senior team member.

We will use study design-specific tools that best account for potential sources of bias. When possible, we will rely on the quality assessments reported in the previous systematic reviews. For these cases, one reviewer will extract the assessments into DistillerSR, and another reviewer will verify the appraisals. Any discrepancies will be resolved by consulting with a senior team member. If items are missing from the adapted risk of bias tools used by the previous review that are needed for subsequent stages of these planned reviews (e.g., grading the certainty of evidence, Task Force evidence to decision table), we may add the original items to the tools or use the original tool and re-assess with two reviewers. The risk of bias assessments for each study will be used to inform the study limitations domain of the certainty of evidence assessment [100].

For KQ1 and KQ2, the Cochrane review by Ilic and colleagues (2021) and UK NSC [87] used different versions of the Cochrane Risk of Bias tool (i.e., the 2011 and 2019 versions). We will follow the UK NSC [87] approach for risk of bias assessments and use the more recent Cochrane Risk of Bias 2.0 tool [101]. The tool does acknowledge potential biases with outcome ascertainment and measurement (e.g., adjudication process with all-cause and prostate cancer mortality). This is useful when considering sticky-diagnosis and/or slippery-linkage bias and control group contamination bias, which has been previously acknowledged in a number of cancer screening publications [102]. We will consider additional factors that could potentially introduce bias, for example, “spin” or misleading reporting, interpretation, or extrapolation of study results. We will judge each item as “yes,” “probably yes,” “probably no,” “no,” and “no information and will judge each overarching domain as “low,” “some concerns,” or “high” regarding the risk of bias judgment according to criteria outlined in the Cochrane Handbook for Systematic Reviews of Interventions [103].

We will use the Cochrane Risk of Bias 2.0 variant for cluster randomized trials and will consider the assessment of identification/recruitment bias which can occur when participants are recruited after the randomization of clusters (or group of individuals). This process could affect the types of recruited participants due to the awareness of intervention and control clusters [104].

The overall risk of bias will be summarized as low risk of bias if all domains were assessed as “low”; some concerns with the risk of bias if at least one domain was assessed as “some concerns” and no other domains were considered “high”; high risk of bias if at least one domain was assessed as “high,” or “some concerns” for multiple domains.

For non-randomized studies, we will use the ROBINS-I (Risk Of Bias In Non-randomized Studies of Interventions) tool [105].

For KQ3, a new risk of bias assessments will not be conducted. We will instead rely on the UK NSC assessments conducted using the Cochrane Risk of Bias 2.0 tool for randomized trials and AMSTAR 2 for systematic reviews [87].

For KQ4, we will follow the 2014 CTFPHC review methodology and use the GRADE Working Group approach to assess the certainty of evidence in the importance of outcomes or values and preferences for stated preference studies (e.g., discrete choice experiments) [88, 106]. We will categorize the overall risk of bias for each study as low, moderate, serious, or critical for each of the four subdomains ((1) selection of participants into the study, (2) completeness of data, (3) measurement instrument, and (4) data analysis). In addition, we will review the estimates reported and provided to study participants compared to our estimates of the benefits and harms of screening, if the information is reported, and how the estimates were presented to study participants. For studies evaluating the effects of decision aids, we will use modified versions of the original Cochrane Risk of Bias Tool for Randomized Controlled Trials (version 1.0) and/or the International Patient Decision Aid Standards instrument (IPDASi) [107, 108].

### Synthesis of included studies

We will describe the study characteristics, participant characteristics, intervention and comparator details, outcome results, and quality appraisals for the included studies in tables. We will transform data from the included studies to ensure consistent presentation and synthesis of the results across studies, where required. Prior to performing a meta-analysis, we will consider clinical (e.g., patient characteristics and PSA levels) and methodological (e.g., study design and risk of bias) heterogeneity of the included studies. After pooling study data, we will assess statistical heterogeneity using the *I*^2^ statistic and Cochran’s *Q* test (threshold *p* value <0.10). For the *I*^2^ statistic, heterogeneity will be considered either low (0–25%), moderate (25–50%), substantial (50–75%), and considerable (>75%) [109–113]. If study data are appropriate for statistical pooling, we will pool studies using the DerSimonian and Laird random-effect methods. If considerable heterogeneity (*I*^2^ >75%) is detected, we will not pool the studies in a meta-analysis, but instead attempt to explain the reasons for heterogeneity through secondary analyses and meta-regression.

If data are available, we will perform separate secondary analyses according to screening interval (KQ1), PSA thresholds (KQ1b), age <55 years (KQ1c), age 55–69 years (KQ1c), age ≥70 years (KQ1c), ethnicity (KQ1d), obesity as defined by study authors (KQ1d), and family history (KQ1d).

When describing the findings narratively, we will present the range of effects and follow guidance on narrative synthesis [114, 115]. For overdiagnosis, we will report the method used to formulate the estimate. Where applicable, we will pool data from randomized controlled trials and controlled clinical trials separately from observational studies.

We will report the relative risk or odds ratio with corresponding 95% confidence intervals. Where various measurement tools are used, we will report the standardized mean difference with 95% confidence intervals. Analyses will be presented separately for each comparison. We will follow GRADE guidance for calculating relative and absolute effects with 95% confidence intervals and absolute risk reduction for the evidence profile tables and summary of findings [116, 117].

For rare events or low event rates (less than 1%), we will use the Peto one-step odds ratio fixed-effect method [118]. When group imbalances exist (e.g., control groups of unequal sizes), a large magnitude of the effect is observed, or when events are more frequent (5 to 10%), the Mantel-Haenszel fixed-effect method will be used [119]. Where data required for analysis is missing, we will contact the authors of the study for additional information by email every 2 weeks for a maximum of three attempts.

We will perform sensitivity analyses to assess the robustness of the results. This may include restricting analyses to studies of low overall risk of bias, different publication types (e.g., removing abstracts only or preprints), or issues as considered in the risk of bias tool. During the conduct of the systematic review, other issues may become apparent that may require examination through sensitivity analyses. These additional analyses are deemed exploratory in nature and should not be construed as a priori with a definitive hypothesis.

We will follow previous guidance on meta-regression analyses when at least 10 studies are available for the outcome and intervention comparisons and this will be based on random-effects models [109]. Funnel plots and statistical tests (e.g., Egger regression test, Hedges-Olkin method, trim-and-fill method) will be used to assess for small-study effects (e.g., publication bias) [111, 120, 121].

### Grading the certainty of evidence and interpretation

The certainty of evidence for all outcomes will be assessed with the Grading of Recommendations, Assessment, Development and Evaluation (GRADE) approach [100, 122]. The GRADE framework is based on five domains: study limitations (risk of bias), inconsistency or data heterogeneity, indirectness of evidence, imprecision of effect size estimates, and risk of publication (small study) bias. The overall assessment will be rated very low, low, moderate, or high for each outcome, and we will assess the certainty of evidence for trials (beginning at high certainty) and non-randomized studies (beginning at low certainty or high if using ROBINS-I tool). Regarding KQ4 (patient values and preferences), we will follow GRADE guidance on assessing the certainty of evidence in the importance of outcomes for values and preferences [106, 123].

Before conducting the assessments, reviewers will pilot GRADE assessments on a sample of five outcomes using GRADEpro GDT online software until reviewer agreement on all domains is high (>95%). Any discrepancies among reviewers will be resolved by consulting with a senior team member. GRADE assessments will be completed independently by pairs of reviewers and any disagreements will be resolved by discussion or consulting a senior team member. The certainty of evidence assessments will also report on the included studies from the previous reviews [87, 88].

For each KQ and the related critical and important outcomes, we will prepare separate GRADE evidence profiles and summary of findings tables with explanations for rating up or down for each domain [116, 117]. When a meta-analysis is not appropriate (e.g., due to considerable heterogeneity), we will follow GRADE guidance on rating the certainty of evidence in the absence of a single estimate of effect [124]. When rating the certainty of the evidence, we will use either a minimally contextualized or partially contextualized approach recommended for systematic review authors [125, 126]. Depending on the approach, we will rate our certainty on whether the true effect lies on one side of the null threshold (i.e., that a non-null effect is present), on one side of a minimally important threshold (i.e., that there is an important versus trivial effect), or within ranges of specific magnitudes (i.e., no or trivial, small, moderate or large effect). The Task Force may choose to fully contextualize the range of possible effects for their Evidence to Decision (EtD) tables. GRADE narrative statements will be prepared to represent the quantity of the evidence, magnitude of effect, and certainty of the evidence [117, 127, 128]. The certainty of evidence assessments for all KQs will be integrated into GRADE EtD tables prepared by the Task Force and Science Team of the Global Health and Guidelines Division at the Public Health Agency of Canada [129]. The EtD framework will include additional information about the intervention (e.g., cost, feasibility) to assist the Task Force in writing updated clinical practice recommendations for screening for prostate cancer.

## Discussion

Since the release of the 2014 Task Force guideline on screening for prostate cancer with the prostate-specific antigen test, there have been new studies and updates to previously included studies. Findings from the planned systematic reviews will inform the Task Force on the update of their recommendations for primary care clinicians on screening for prostate cancer in adults.

## Supplementary Information

**Additional file 1.** Prostate cancer screening guidelines released since 2015.

**Additional file 2.** PRISMA-P 2015 checklist.

**Additional file 3.** Search strategies.

**Additional file 4.** PRESS checklist.

**Additional file 5.** List of grey literature sources.

**Additional file 6.** Draft data extraction items.

**Additional file 7.** Stakeholder review and feedback.

## Data Availability

Not applicable.

## Abbreviations

CI: Confidence interval
DRE: Digital rectal examination
ERSC: Evidence Review and Synthesis Center
ERSPC: European Randomized Study of Screening for Prostate Cancer
GRADE: Grading of Recommendations Assessment, Development and Evaluation
KQ: Key question
PSA: Prostate-specific antigen
UK NSC: UK National Screening Committee
USPSTF: United States Preventive Services Task Force
