# Supplementary material for: Screening for prostate cancer: protocol for updating multiple systematic reviews to inform a Canadian Task Force on Preventive Health Care guideline update
Source: Syst Rev. 2022 Oct 26;11:230. doi: 10.1186/s13643-022-02099-9 (PMC9609189; doi:10.1186/s13643-022-02099-9)
Supplement: Supplementary file 1 — Additional file 1. Prostate cancer screening guidelines released since 2015. [file 13643_2022_2099_MOESM1_ESM.docx]

## Additional file 1: Prostate cancer screening guidelines released since 2015

| **2022** | **Canadian Urological Association:** <https://www.cua.org/program/10892>   - Login required to view recommendations |
| --- | --- |
| **2020** | **European Association of Urology (EAU)-European Association of Nuclear Medicine (EANM)-European Society for Radiotherapy and Oncology (ESTRO)-European Society of Urogenital Radiology (ESUR)-International Society of Geriatric Oncology (SIOG) guidelines on screening, diagnosis, and local treatment of clinically localised prostate cancer (PCa).:** <https://www.europeanurology.com/article/S0302-2838(20)30769-7/fulltext>  Recommendations:   1. Do not subject men to prostate-specific antigen (PSA) testing without counselling them on the potential risks and benefits, Strength rating: Strong 2. Offer an individualised risk-adapted strategy for early detection to a well-informed man with life-expectancy of at least 10–15 yr, Strength rating: Weak 3. Offer early PSA testing to well-informed men at an elevated risk of having PCa:    1. Men >50 yr of age    2. Men >45 yr of age with a family history of PCa    3. Men of African descent >45 yr of age    4. Men carrying BRCA2 mutations >40 yr of age   Strength rating: Strong   1. Offer a risk-adapted strategy (based on initial PSA level), with follow-up intervals of 2 yr for those initially at risk:    1. Men with a PSA level of >1 ng/mL at 40 yr of age    2. Men with a PSA level of >2 ng/mL at 60 yr of age   Postpone follow-up to 8 yr in those not at risk, Strength rating: Weak   1. Stop early diagnosis of PCa based on life expectancy and performance status; men who have life expectancy of <15 yr are unlikely to benefit, Strength rating: Strong |
| **2018** | **US Preventive Services Task Force:** <https://pubmed.ncbi.nlm.nih.gov/29801017/>   - *Provide information about potential benefits and harms of screening for men aged 55-69 years. Recommend against screening for men and => 70 years (Weak recommendation; low-moderate evidence)*   **US National Comprehensive Cancer Network**: <https://www.nccn.org/professionals/physician_gls/default.aspx>   - Login required to view recommendations   **European Association of Urology**: <https://uroweb.org/guideline/prostate-cancer/>   - Login required to view recommendations   **American Urological Association**, reviewed and validity confirmed in 2018: <https://www.auanet.org//guidelines-and-quality/guidelines/prostate-cancer-early-detection-guideline>   - The panel recommends against screening for men <55 years at average risk, men ≥70 years, or men with less than a 10 to 15-year life expectancy - *“For men ages 55 to 69 years the Panel recognizes that the decision to undergo PSA screening involves weighing the benefits of reducing the rate of metastatic prostate cancer and prevention of prostate cancer death against the known potential harms associated with screening and treatment. For this reason, the Panel strongly recommends shared decision-making for men age 55 to 69 years that are considering PSA screening, and proceeding based on a man's values and preferences. (Standard; Evidence Strength Grade B)”*   **Members of the Rapid Recommendation panel:** <https://www.bmj.com/content/362/bmj.k3581>   - *“We suggest against systematic PSA-based screening for prostate cancer. Either option is reasonable. Shared decision making is needed for men considering screening.”* |
| **2017** | **US Memorial Sloan Kettering Cancer Center:** <https://www.mskcc.org/cancer-care/types/prostate/screening/screening-guidelines-prostate>   - Recommendations for men ages:   - 45 to 49 should have a baseline PSA test.   - 50 to 70 should have their PSA level checked.   - 71 to 75 should talk with their doctor about whether to have a PSA test. This decision should be based on past PSA levels and the health of the man.   - ≥76 years prostate cancer screening is not recommended   (No information provided on level of evidence or recommendation)  **Cancer Council Australia/Prostate Cancer Foundation of Australia:** <https://www.prostate.org.au/awareness/for-healthcare-professionals/clinical-practice-guidelines-on-psa-testing/>   - *“The guideline does not recommend a population screening program for prostate cancer (a program that offers testing to all men in a certain age group who do not have prostate cancer or symptoms that suggest prostate cancer).”*   **Canadian Urological Association:** <https://www.ncbi.nlm.nih.gov/pmc/articles/PMC5659858/pdf/cuaj-10-298.pdf>   - Offer PSA screening to men with a life expectancy >10 years. The decision of whether to pursue PSA screening should be based on shared decision-making after the potential benefits and harms associated with screening have been discussed (Level of evidence: 1; Grade of recommendation: B). |
| **2016** | **American Cancer Society (last medical review August 1, 2019)**: <https://www.cancer.org/cancer/prostate-cancer/detection-diagnosis-staging/acs-recommendations.html>   - *“The American Cancer Society (ACS) recommends that men have a chance to make an informed decision with their health care provider about whether to be screened for prostate cancer. The decision should be made after getting information about the uncertainties, risks, and potential benefits of prostate cancer screening.”*   **UK National Screening Committee:** <https://legacyscreening.phe.org.uk/prostatecancer>   - A *“systematic population screening programme [is] not recommended”* |
| **2015** | **European Society for Medical Oncology**: <https://www.ncbi.nlm.nih.gov/pubmed/26205393>   - *“Population-based PSA screening for prostate cancer reduces prostate cancer mortality at the expense of over diagnosis and overtreatment and is not recommended [I, C].* - *Testing for prostate cancer in asymptomatic men should not be done in men over the age of 70 years [I, B].”*   **U.S. National Comprehensive Cancer Network**: <https://jnccn.org/view/journals/jnccn/13/12/article-p1534.xml>   - *“The panel recommends that baseline PSA testing should be offered to healthy, well-informed men aged 45 to 75 years based on the results of RCTs. Baseline testing may be complemented by DRE.”* |
