# Supplementary material for: Screening for prostate cancer: protocol for updating multiple systematic reviews to inform a Canadian Task Force on Preventive Health Care guideline update
Source: Syst Rev. 2022 Oct 26;11:230. doi: 10.1186/s13643-022-02099-9 (PMC9609189; doi:10.1186/s13643-022-02099-9)
Supplement: Supplementary file 3 — Additional file 3. Search strategies. [file 13643_2022_2099_MOESM3_ESM.docx]

## Additional file 3: Search strategies

### Key question 1 and 2

UK NSC, Ilic Updated

2020 Nov 13

Ovid Multifile

Database: Embase Classic+Embase <1947 to 2020 November 12> , Ovid MEDLINE(R) ALL <1946 to November 12, 2020>, EBM Reviews - Cochrane Central Register of Controlled Trials <October 2020>

Search Strategy:

--------------------------------------------------------------------------------

1 exp Prostatic Neoplasms/ (390321)

2 (prostat* adj3 (neoplas* or cancer* or carcinoma* or adenocarcinom* or tumour* or tumor* or malignan* or metasta* or angiosarcoma* or sarcoma* or teratoma* or lymphoma* or blastoma* or microcytic* or leiomyosarcoma* or lump?)).tw,kf. (389915)

3 (PIN and (prostat* or intraepithelial or neoplas*)).tw,kf. (3384)

4 or/1-3 [PROSTATE CANCER] (468552)

5 Mass Screening/ (169100)

6 "Early Detection of Cancer"/ (33130)

7 (screen* or detect*).tw,kf. (7111817)

8 (identif* or recogni*).ti. (874161)

9 ((early or earlier or earliest) adj5 (identif* or recogni*)).tw,kf. (186852)

10 (case finding? or casefinding?).tw,kf. (13624)

11 exp Prostatic Neoplasms/di, pc [Diagnosis, Prevention & Control] (60956)

12 Prostatic Intraepithelial Neoplasia/di, pc [Diagnosis, Prevention & Control] (838)

13 Digital Rectal Examination/ (10044)

14 (rect* adj2 exam*).tw,kf. (18098)

15 DRE.tw,kf. (8277)

16 Palpation/ (28873)

17 palpat*.tw,kf. (43273)

18 ((transrectal* or trans-rectal*) adj4 (ultrasonograph* or ultrasound* or ultra-sonograph* or ultra-sound*)).tw,kf. (21766)

19 (TRUS or TRUSB).tw,kf. (10000)

20 ((prostat* specific antigen* or PSA) adj3 (diagnos* or screen* or test*)).tw,kf. (19955)

21 Prostate-Specific Antigen/ and (Diagnostic Tests, Routine/ or "Diagnostic Techniques and Procedures"/) (1033)

22 or/5-21 [SCREENING] (8062065)

23 4 and 22 [PROSTATE CANCER - SCREENING] (148972)

24 (controlled clinical trial or randomized controlled trial or pragmatic clinical trial or equivalence trial).pt. (1200351)

25 "Clinical Trials as Topic"/ (306938)

26 exp "Controlled Clinical Trials as Topic"/ (353440)

27 (randomi#ed or randomi#ation? or randomly or RCT or placebo*).tw,kf. (3520109)

28 ((singl* or doubl* or trebl* or tripl*) adj (mask* or blind* or dumm*)).tw,kf. (707249)

29 trial.ti. (884282)

30 or/24-29 (4450597)

31 23 and 30 [PROSTATE CANCER - SCREENING - RCTs] (15469)

32 (2019082* or 2019083* or 201909* or 201910* or 201911* or 201912* or 202*).dt. (1742359)

33 31 and 32 [RCTs - UPDATE PERIOD] (262)

34 controlled clinical trial.pt. (185685)

35 Controlled Clinical Trial/ or Controlled Clinical Trials as Topic/ (576181)

36 (control* adj2 trial).tw,kf. (642455)

37 Non-Randomized Controlled Trials as Topic/ (12100)

38 (nonrandom* or non-random* or quasi-random* or quasi-experiment*).tw,kf. (147536)

39 (nRCT or non-RCT).tw,kf. (904)

40 Controlled Before-After Studies/ (221763)

41 (control* adj3 ("before and after" or "before after")).tw,kf. (769107)

42 Interrupted Time Series Analysis/ (216906)

43 time series.tw,kf. (70939)

44 (pre- adj3 post-).tw,kf. (273030)

45 (pretest adj3 posttest).tw,kf. (15369)

46 Historically Controlled Study/ (232193)

47 (control* adj2 study).tw,kf. (529642)

48 Control Groups/ (125056)

49 (control* adj2 group?).tw,kf. (1501236)

50 trial.ti. (884282)

51 or/34-50 (4174146)

52 23 and 51 [PROSTATE CANCER - SCREENING - nRCTs] (12622)

53 (201206* or 201207* or 201208* or 201209* or 201210* or 201211* or 201212* or 2013* or 2014* or 2015* or 2016* or 2017* or 2018* or 2019* or 202*).dt. (9968146)

54 52 and 53 [nRCTs - UPDATE PERIOD] (1543)

55 33 or 54 [RCTS, nRCTs] (1677)

56 exp Animals/ not Humans/ (18341314)

57 55 not 56 [ANIMAL-ONLY REMOVED] (1659)

58 (comment or editorial or news or newspaper article).pt. (2136071)

59 (letter not (letter and randomized controlled trial)).pt. (2257686)

60 57 not (58 or 59) [OPINION PIECES REMOVED] (1571)

61 60 use medall [MEDLINE RECORDS] (1571)

62 exp prostate tumor/ (254992)

63 (prostat* adj3 (neoplas* or cancer* or carcinoma* or adenocarcinom* or tumour* or tumor* or malignan* or metasta* or angiosarcoma* or sarcoma* or teratoma* or lymphoma* or blastoma* or microcytic* or leiomyosarcoma* or lump?)).tw,kw. (393052)

64 (PIN and (prostat* or intraepithelial or neoplas*)).tw,kw. (3437)

65 or/62-64 [PROSTATE CANCER] (447538)

66 mass screening/ (169100)

67 cancer screening/ (108144)

68 early cancer diagnosis/ (7373)

69 (screen* or detect*).tw,kw. (7126232)

70 (identif* or recogni*).ti. (874161)

71 ((early or earlier or earliest) adj5 (identif* or recogni*)).tw,kw. (186978)

72 (case finding? or casefinding?).tw,kw. (13779)

73 exp prostate tumor/di, pc [Diagnosis, Prevention & Control] (38637)

74 digital rectal examination/ (10044)

75 (rect* adj2 exam*).tw,kw. (18220)

76 DRE.tw,kw. (8299)

77 palpation/ (28873)

78 palpat*.tw,kw. (43475)

79 transrectal ultrasonography/ (12751)

80 ((transrectal* or trans-rectal*) adj4 (ultrasonograph* or ultrasound* or ultra-sonograph* or ultra-sound*)).tw,kw. (21946)

81 (TRUS or TRUSB).tw,kw. (10041)

82 ((prostat* specific antigen* or PSA) adj3 (diagnos* or screen* or test*)).tw,kw. (20131)

83 prostate specific antigen/ and (diagnostic test/ or laboratory test/) (1170)

84 or/66-83 [SCREENING] (8081913)

85 65 and 84 [PROSTATE CANCER - SCREENING] (136699)

86 exp randomized controlled trial/ or controlled clinical trial/ (1431519)

87 clinical trial/ (1540585)

88 exp "controlled clinical trial (topic)"/ (198780)

89 (randomi#ed or randomi#ation? or randomly or RCT or placebo*).tw,kw. (3581560)

90 ((singl* or doubl* or trebl* or tripl*) adj (mask* or blind* or dumm*)).tw,kw. (735058)

91 trial.ti. (884282)

92 or/86-91 [RCT FILTER] (5041980)

93 85 and 92 [PROSTATE CANCER - SCREENING - RCTs] (18576)

94 (2019082* or 2019083* or 201909* or 201910* or 201911* or 201912* or 202*).dc. (2657362)

95 93 and 94 [RCTs - UPDATE PERIOD] (892)

96 exp controlled clinical trial/ (1431415)

97 exp "controlled clinical trial (topic)"/ (198780)

98 (control* adj2 trial*).kw,tw. (1273841)

99 (nonrandom* or non-random* or quasi-random* or quasi-experiment*).kw,tw. (148390)

100 (nRCT or non-RCT).kw,tw. (905)

101 (control* adj3 ("before and after" or "before after")).kw,tw. (769112)

102 time series analysis/ (27698)

103 time series.kw,tw. (71939)

104 pretest posttest control group design/ (509)

105 (pre- adj3 post-).kw,tw. (273075)

106 (pretest adj3 posttest).kw,tw. (18542)

107 controlled study/ (7834517)

108 (control* adj2 stud$3).kw,tw. (1065218)

109 control group/ (124958)

110 (control$ adj2 group$1).kw,tw. (1502249)

111 or/96-110 (11206368)

112 or/96-110 [nRCT FILTER] (11206368)

113 85 and 112 [PROSTATE CANCER - SCREENING - nRCTs] (36513)

114 (201206* or 201207* or 201208* or 201209* or 201210* or 201211* or 201212* or 2013* or 2014* or 2015* or 2016* or 2017* or 2018* or 2019* or 202*).dc. (14322013)

115 113 and 114 [nRCTs - UPDATE PERIOD] (18157)

116 95 or 115 [RCTs, nRCTs] (18307)

117 exp animal/ or exp animal experimentation/ or exp animal model/ or exp animal experiment/ or nonhuman/ or exp vertebrate/ (54624044)

118 exp human/ or exp human experimentation/ or exp human experiment/ (42458596)

119 117 not 118 (12167259)

120 116 not 119 [ANIMAL-ONLY REMOVED] (17450)

121 editorial.pt. (1222781)

122 letter.pt. not (letter.pt. and randomized controlled trial/) (2257520)

123 120 not (121 or 122) [OPINION PIECES REMOVED] (17309)

124 123 use emczd [EMBASE RECORDS] (17309)

125 exp Prostatic Neoplasms/ (390321)

126 (prostat* adj3 (neoplas* or cancer* or carcinoma* or adenocarcinom* or tumour* or tumor* or malignan* or metasta* or angiosarcoma* or sarcoma* or teratoma* or lymphoma* or blastoma* or microcytic* or leiomyosarcoma* or lump?)).ti,ab,kw. (393052)

127 (PIN and (prostat* or intraepithelial or neoplas*)).ti,ab,kw. (3437)

128 or/125-127 [PROSTATE CANCER] (469956)

129 Mass Screening/ (169100)

130 "Early Detection of Cancer"/ (33130)

131 (screen* or detect*).ti,ab,kw. (7126224)

132 (identif* or recogni*).ti. (874161)

133 ((early or earlier or earliest) adj5 (identif* or recogni*)).ti,ab,kw. (186978)

134 (case finding? or casefinding?).ti,ab,kw. (13779)

135 exp Prostatic Neoplasms/di, pc [Diagnosis, Prevention & Control] (60956)

136 Prostatic Intraepithelial Neoplasia/di, pc [Diagnosis, Prevention & Control] (838)

137 Digital Rectal Examination/ (10044)

138 (rect* adj2 exam*).ti,ab,kw. (18220)

139 DRE.ti,ab,kw. (8299)

140 Palpation/ (28873)

141 palpat*.ti,ab,kw. (43475)

142 ((transrectal* or trans-rectal*) adj4 (ultrasonograph* or ultrasound* or ultra-sonograph* or ultra-sound*)).ti,ab,kw. (21946)

143 (TRUS or TRUSB).ti,ab,kw. (10041)

144 ((prostat* specific antigen* or PSA) adj3 (diagnos* or screen* or test*)).ti,ab,kw. (20131)

145 Prostate-Specific Antigen/ and (Diagnostic Tests, Routine/ or "Diagnostic Techniques and Procedures"/) (1033)

146 or/129-145 [SCREENING] (8076142)

147 128 and 146 [PROSTATE CANCER - SCREENING] (149460)

148 (2019082* or 2019083* or 201909* or 201910* or 201911* or 201912* or 202*).up. (35268983)

149 147 and 148 [RCTs - UPDATE PERIOD] (62220)

150 149 use cctr [CENTRAL RECORDS] (1321)

151 61 or 124 or 150 [ALL DATABASES] (20201)

152 (conference abstract or journal conference abstract).pt. (4083807)

153 151 not 152 [CONFERENCE ABSTRACTS REMOVED] (14151)

154 limit 153 to yr="2018-current" (5740)

155 remove duplicates from 154 (5059)

156 limit 153 to yr="2014-2017" (5903)

157 remove duplicates from 156 (5217)

158 153 not (154 or 156) (2508)

159 remove duplicates from 158 (2291)

160 155 or 157 or 159 [TOTAL UNIQUE RECORDS] (12567)

161 160 use medall [MEDLINE UNIQUE RECORDS] (1565)

162 160 use emczd [EMBASE UNIQUE RECORDS] (10069)

163 160 use cctr [CENTRAL UNIQUE RECORDS] (933)

### Key question 4

Vernooji – Updated

2020 Nov 28

Ovid Multifile

Database: Embase Classic+Embase <1947 to 2020 November 25> , Ovid MEDLINE(R) ALL <1946 to November 25, 2020>, APA PsycInfo <1806 to November Week 3 2020>

Search Strategy:

--------------------------------------------------------------------------------

1 exp Prostatic Neoplasms/ (385550)

2 (prostat* adj3 (neoplas* or cancer* or carcinoma* or adenocarcinom* or tumour* or tumor* or malignan* or metasta* or angiosarcoma* or sarcoma* or teratoma* or lymphoma* or blastoma* or microcytic* or leiomyosarcoma* or lump?)).tw,kf. (380222)

3 (PIN and (prostat* or intraepithelial or neoplas*)).tw,kf. (3335)

4 or/1-3 [PROSTATE CANCER] (458504)

5 Mass Screening/ (166250)

6 "Early Detection of Cancer"/ (32117)

7 (screen* or detect*).tw,kf. (7212547)

8 (identif* or recogni*).ti. (929952)

9 ((early or earlier or earliest) adj5 (identif* or recogni*)).tw,kf. (198815)

10 (case finding? or casefinding?).tw,kf. (14103)

11 exp Prostatic Neoplasms/di, pc [Diagnosis, Prevention & Control] (61095)

12 Prostatic Intraepithelial Neoplasia/di, pc [Diagnosis, Prevention & Control] (823)

13 Digital Rectal Examination/ (10031)

14 (rect* adj2 exam*).tw,kf. (17514)

15 DRE.tw,kf. (8122)

16 Palpation/ (28560)

17 palpat*.tw,kf. (41607)

18 ((prostat* specific antigen* or PSA) adj3 (diagnos* or screen* or test*)).tw,kf. (19051)

19 Prostate-Specific Antigen/ and (Diagnostic Tests, Routine/ or "Diagnostic Techniques and Procedures"/) (1035)

20 or/5-19 [SCREENING] (8207516)

21 4 and 20 [PROSTATE CANCER - SCREENING] (142386)

22 exp Prostatic Neoplasms/px [Psychology] (2472)

23 Mass Screening/px [Psychology] (2384)

24 "Early Detection of Cancer"/px [Psychology] (1361)

25 Attitude/ (118727)

26 Attitude to Death/ (27404)

27 exp Attitude to Health/ (541583)

28 Choice Behavior/ (266383)

29 Consumer Advocacy/ (6646)

30 *Consumer Behavior/ (38640)

31 Cooperative Behavior/ (81956)

32 Decision Making/ (413220)

33 Decision Support Techniques/ (40402)

34 Focus Groups/ (231036)

35 Health Care Surveys/ (45541)

36 Health Services Accessibility/ (127242)

37 Interviews as Topic/ (225675)

38 Life Change Events/ (50019)

39 Narration/ (23465)

40 Patient Acceptance of Health Care/ (108248)

41 Patient Advocacy/ (45990)

42 exp Patient-Centered Care/ (873644)

43 exp Patient Education as Topic/ (201398)

44 Patient Participation/ (56937)

45 Patient Preference/ (28405)

46 Patient Satisfaction/ (230838)

47 Patients/px (8472)

48 Personal Autonomy/ (31114)

49 *"Power (Psychology)"/ (69391)

50 Quality of Life/px (28027)

51 Questionnaires/ (1091058)

52 exp Self Concept/ (400998)

53 Self Efficacy/ (104321)

54 exp Self-Help Groups/ (24482)

55 Social Values/ (106436)

56 Uncertainty/ (54778)

57 (choice or choices or choose or chooses or chose or chosen).ti. (124982)

58 prefer*.ti. (133318)

59 ((accept* or anxiet* or anxious* or attitud* or consider* or choice? or choos* or chose? or concern$2 or decid* or decis* or dissatisf* or expect* or experienc* or fear* or feel* or felt or input* or knowledge* or opinion* or participat* or perceiv* or percepti* or perspective? or prefer* or "point-of-view" or "points-of-view" or respons* or satisf* or uncertain* or understand* or unsatisf* or valuation* or value? or valuing or view or views or viewpoint* or worrie? or worry*) adj3 (male? or men or patient or patients or patient-oriented or patient-related or person$2 or personally or user or users or user-oriented or user-related)).tw,kf. (2166294)

60 ((accept* or anxiet* or anxious* or attitud* or consider* or choice? or choos* or chose? or concern$2 or decid* or decis* or dissatisf* or expect* or experienc* or fear* or feel* or felt or input* or knowledge* or opinion* or participat* or perceiv* or percepti* or perspective? or prefer* or "point-of-view" or "points-of-view" or respons* or satisf* or uncertain* or understand* or unsatisf* or valuation* or value? or valuing or view or views or viewpoint* or worrie? or worry*) adj3 (death or health* or life or living)).tw,kf. (1019288)

61 ((analys#s or valuation? or value? or valuing) adj3 (conjoint or contingent)).tw,kf. (5203)

62 best worst.tw,kf. (1131)

63 (choice? adj2 behavio?r*).tw,kf. (13128)

64 (choice? adj1 (discrete or experiment*)).tw,kf. (10303)

65 (decision* adj3 (analy* or board?)).tw,kf. (40728)

66 (decision* adj3 (making or makes or made or undertak*)).tw,kf. (508523)

67 (decision* adj3 (aid or aids or support* or tool?)).tw,kf. (93568)

68 ((emotion? or feeling?) adj3 thermometer?).tw,kf. (365)

69 empower*.tw,kf. (94210)

70 (focus group? or interview* or questionnaire? or survey*).tw,kf. (4030166)

71 (freedom? or libert*).tw,kf. (146124)

72 gambl*.tw,kf. (35971)

73 informed choice?.tw,kf. (6994)

74 (life adj3 (event? or experience?)).tw,kf. (117136)

75 (multi?attribute or multi?criteria).tw,kf. (3393)

76 (preference? adj1 (elicit* or scor* or stated)).tw,kf. (5550)

77 prospect theor*.tw,kf. (1840)

78 ((person$2 or self) adj2 (conceiv* or concept*)).tw,kf. (48593)

79 ((person$2 or self) adj2 (determin* or efficac* or help or manag* or support*)).tw,kf. (257227)

80 (social* adj1 valu*).tw,kf. (9844)

81 exp Communication/ (1316723)

82 ((time$2 or timeliness) adj2 (communica* or info*)).tw,kf. (23744)

83 (miscommunicat* or mis-communicat*).tw,kf. (3053)

84 (misunderstand* or mis-understand*).tw,kf. (20592)

85 (misinform* or mis-inform*).tw,kf. (9831)

86 ((influenc* or influential or involv*) adj3 (male? or men or patient or patients or patient-oriented or patient-related or person$2 or personally or user or users or user-oriented or user-related)).tw,kf. (301501)

87 exp Informed Consent/ (156561)

88 (informed adj3 (choice* or choos* or consent* or decid* or decision*)).tw,kf. (169945)

89 (consent* adj3 (male? or men or patient or patients or patient-oriented or patient-related or person$2 or personally or user or users or user-oriented or user-related)).tw,kf. (49806)

90 ((guide? or guiding or influenc* or influential or make or making or makes or made or shar* or support*) adj2 (choice? or choos* or consent* or decid* or decision*)).tw,kf. (645306)

91 (screen* adj3 (choice? or choos* or consent* or decid* or decision*)).tw,kf. (10471)

92 or/22-91 [PATIENT PREFERENCES/VALUES/QOL] (10581364)

93 21 and 92 [PROSTATE CANCER - PATIENT PREFERENCES/VALUES/QOL] (27714)

94 exp Animals/ not Humans/ (18790011)

95 93 not 94 [ANIMAL-ONLY REMOVED] (20078)

96 (201709* or 201710* or 201711* or 201712* or 2018* or 2019* or 2020* or 2021*).dt. (4319900)

97 95 and 96 [UPDATE PERIOD] (1669)

98 97 use medall [MEDLINE RECORDS] (1669)

99 exp prostate tumor/ (255584)

100 (prostat* adj3 (neoplas* or cancer* or carcinoma* or adenocarcinom* or tumour* or tumor* or malignan* or metasta* or angiosarcoma* or sarcoma* or teratoma* or lymphoma* or blastoma* or microcytic* or leiomyosarcoma* or lump?)).tw,kw. (382776)

101 (PIN and (prostat* or intraepithelial or neoplas*)).tw,kw. (3387)

102 or/99-101 [PROSTATE CANCER] (437397)

103 mass screening/ (166250)

104 cancer screening/ (112107)

105 early cancer diagnosis/ (7460)

106 (screen* or detect*).tw,kw. (7224088)

107 (identif* or recogni*).ti. (929952)

108 ((early or earlier or earliest) adj5 (identif* or recogni*)).tw,kw. (198939)

109 (case finding? or casefinding?).tw,kw. (14248)

110 exp prostate tumor/di, pc [Diagnosis, Prevention] (38746)

111 digital rectal examination/ (10031)

112 (rect* adj2 exam*).tw,kw. (17555)

113 DRE.tw,kw. (8143)

114 palpation/ (28560)

115 palpat*.tw,kw. (41751)

116 ((prostat* specific antigen* or PSA) adj3 (diagnos* or screen* or test*)).tw,kw. (19200)

117 prostate specific antigen/ and (diagnostic test/ or laboratory test/) (1173)

118 or/103-117 [SCREENING] (8222882)

119 102 and 118 [PROSTATE CANCER - SCREENING] (129697)

120 adaptive behavior/ (153547)

121 attitude/ (118727)

122 attitude to death/ (27404)

123 attitude to disability/ (356)

124 attitude to health/ (201313)

125 attitude to illness/ (5969)

126 attitude to life/ (740)

127 consumer advocacy/ (6646)

128 consumer attitude/ (20300)

129 cooperation/ (59091)

130 decision making/ (413220)

131 health care survey/ (50933)

132 exp interview/ (327479)

133 life event/ (29724)

134 patient advocacy/ (45990)

135 exp patient attitude/ (437949)

136 patient decision making/ (10354)

137 exp patient education/ (205490)

138 personal autonomy/ (31114)

139 psychological aspect/ (483964)

140 exp questionnaire/ (1822968)

141 exp self concept/ (400998)

142 self help/ (18459)

143 exp social psychology/ (1040429)

144 uncertainty/ (54778)

145 (choice or choices or choose or chooses or chose or chosen).ti. (124982)

146 prefer*.ti. (133318)

147 ((accept* or anxiet* or anxious* or attitud* or consider* or choice? or choos* or chose? or concern$2 or decid* or decis* or dissatisf* or expect* or experienc* or fear* or feel* or felt or input* or knowledge* or opinion* or participat* or perceiv* or percepti* or perspective? or prefer* or "point-of-view" or "points-of-view" or respons* or satisf* or uncertain* or understand* or unsatisf* or valuation* or value? or valuing or view or views or viewpoint* or worrie? or worry*) adj3 (male? or men or patient or patients or patient-oriented or patient-related or person$2 or personally or user or users or user-oriented or user-related)).tw,kw. (2168098)

148 ((accept* or anxiet* or anxious* or attitud* or consider* or choice? or choos* or chose? or concern$2 or decid* or decis* or dissatisf* or expect* or experienc* or fear* or feel* or felt or input* or knowledge* or opinion* or participat* or perceiv* or percepti* or perspective? or prefer* or "point-of-view" or "points-of-view" or respons* or satisf* or uncertain* or understand* or unsatisf* or valuation* or value? or valuing or view or views or viewpoint* or worrie? or worry*) adj3 (death or health* or life or living)).tw,kw. (1025953)

149 ((analys#s or valuation? or value? or valuing) adj3 (conjoint or contingent)).tw,kw. (5337)

150 best worst.tw,kw. (1135)

151 (choice? adj2 behavio?r*).tw,kw. (13286)

152 (choice? adj1 (discrete or experiment*)).tw,kw. (10366)

153 (decision* adj3 (analy* or board?)).tw,kw. (41166)

154 (decision* adj3 (making or makes or made or undertak*)).tw,kw. (511099)

155 (decision* adj3 (aid or aids or support* or tool?)).tw,kw. (94621)

156 ((emotion? or feeling?) adj3 thermometer?).tw,kw. (365)

157 empower*.tw,kw. (94805)

158 (focus group? or interview* or questionnaire? or survey*).tw,kw. (4033371)

159 (freedom? or libert*).tw,kw. (146232)

160 gambl*.tw,kw. (36133)

161 informed choice?.tw,kw. (7100)

162 (life adj3 (event? or experience?)).tw,kw. (117450)

163 (multi?attribute or multi?criteria).tw,kw. (3492)

164 (preference? adj1 (elicit* or scor* or stated)).tw,kw. (5585)

165 prospect theor*.tw,kw. (1891)

166 ((person$2 or self) adj2 (conceiv* or concept*)).tw,kw. (48613)

167 ((person$2 or self) adj2 (determin* or efficac* or help or manag* or support*)).tw,kw. (258203)

168 (social* adj1 valu*).tw,kw. (10198)

169 communication/ (244099)

170 exp verbal communication/ (472343)

171 ((time$2 or timeliness) adj2 (communica* or info*)).tw,kw. (23773)

172 (miscommunicat* or mis-communicat*).tw,kw. (3069)

173 (misunderstand* or mis-understand*).tw,kw. (20606)

174 (misinform* or mis-inform*).tw,kw. (9907)

175 ((influenc* or influential or involv*) adj3 (male? or men or patient or patients or patient-oriented or patient-related or person$2 or personally or user or users or user-oriented or user-related)).tw,kw. (301642)

176 exp Informed Consent/ (156561)

177 (informed adj3 (choice* or choos* or consent* or decid* or decision*)).tw,kw. (170998)

178 (consent* adj3 (male? or men or patient or patients or patient-oriented or patient-related or person$2 or personally or user or users or user-oriented or user-related)).tw,kw. (49848)

179 ((guide? or guiding or influenc* or influential or make or making or makes or made or shar* or support*) adj2 (choice? or choos* or consent* or decid* or decision*)).tw,kw. (648472)

180 (screen* adj3 (choice? or choos* or consent* or decid* or decision*)).tw,kw. (10560)

181 or/120-180 [PATIENT PREFERENCES/VALUES/QOL] (10551962)

182 119 and 181 [PROSTATE CANCER - PATIENT PREFERENCES/VALUES/QOL] (25435)

183 exp animal/ or exp animal experimentation/ or exp animal model/ or exp animal experiment/ or nonhuman/ or exp vertebrate/ (54480830)

184 exp human/ or exp human experimentation/ or exp human experiment/ (41958606)

185 183 not 184 (12524036)

186 182 not 185 [ANIMAL-ONLY REMOVED] (25325)

187 (201709* or 201710* or 201711* or 201712* or 2018* or 2019* or 2020* or 2021*).dc. (6054254)

188 186 and 187 [UPDATE PERIOD] (4044)

189 conference abstract.pt. (3913028)

190 188 not 189 [CONFERENCE ABSTRACTS REMOVED] (2430)

191 190 use emczd [EMBASE RECORDS] (2430)

192 Prostate/ and exp Neoplasms/ (50109)

193 (prostat* adj3 (neoplas* or cancer* or carcinoma* or adenocarcinom* or tumour* or tumor* or malignan* or metasta* or angiosarcoma* or sarcoma* or teratoma* or lymphoma* or blastoma* or microcytic* or leiomyosarcoma* or lump?)).tw,id. (378109)

194 (PIN and (prostat* or intraepithelial or neoplas*)).tw,id. (3318)

195 or/192-194 [PROSTATE CANCER] (391310)

196 Screening/ (302527)

197 Health Screening/ (64803)

198 Cancer Screening/ (112107)

199 (screen* or detect*).tw,id. (7203172)

200 (identif* or recogni*).ti. (929952)

201 ((early or earlier or earliest) adj5 (identif* or recogni*)).tw,id. (198701)

202 (case finding? or casefinding?).tw,id. (14016)

203 (rect* adj2 exam*).tw,id. (17458)

204 DRE.tw,id. (8065)

205 palpat*.tw,id. (41518)

206 ((prostat* specific antigen* or PSA) adj3 (diagnos* or screen* or test*)).tw,id. (19019)

207 or/196-206 [SCREENING] (8184937)

208 195 and 207 [PROSTATE CANCER - SCREENING] (101591)

209 Attitudes/ (75422)

210 Client Attitudes/ (17389)

211 Death Attitudes/ (19686)

212 Explicit Attitudes/ (354)

213 exp Health Attitudes/ (434962)

214 exp Implicit Attitudes/ (1265)

215 "Physical Illness (Attitudes Toward)"/ (2501)

216 Choice Behavior/ (266383)

217 *Consumer Behavior/ (38640)

218 Cooperation/ (59091)

219 Decision Making/ (413220)

220 exp Focus Group/ (1862793)

221 Health Care Access/ (65802)

222 Interviews/ (225803)

223 Life Changes/ (9482)

224 Life Experiences/ (49647)

225 Narratives/ (20425)

226 Advocacy/ (5353)

227 Patient Centered Care/ (213383)

228 Health Education/ (177918)

229 Client Participation/ (2340)

230 Client Satisfaction/ (5648)

231 Preferences/ (17590)

232 Questionnaires/ (1091058)

233 exp Self-Concept/ (400998)

234 Self-Efficacy/ (104321)

235 exp Self-Help Techniques/ (11093)

236 Social Values/ (106436)

237 (choice or choices or choose or chooses or chose or chosen).ti. (124982)

238 prefer*.ti. (133318)

239 ((accept* or anxiet* or anxious* or attitud* or consider* or choice? or choos* or chose? or concern$2 or decid* or decis* or dissatisf* or expect* or experienc* or fear* or feel* or felt or input* or knowledge* or opinion* or participat* or perceiv* or percepti* or perspective? or prefer* or "point-of-view" or "points-of-view" or respons* or satisf* or uncertain* or understand* or unsatisf* or valuation* or value? or valuing or view or views or viewpoint* or worrie? or worry*) adj3 (male? or men or patient or patients or patient-oriented or patient-related or person$2 or personally or user or users or user-oriented or user-related)).tw,id. (2163278)

240 ((accept* or anxiet* or anxious* or attitud* or consider* or choice? or choos* or chose? or concern$2 or decid* or decis* or dissatisf* or expect* or experienc* or fear* or feel* or felt or input* or knowledge* or opinion* or participat* or perceiv* or percepti* or perspective? or prefer* or "point-of-view" or "points-of-view" or respons* or satisf* or uncertain* or understand* or unsatisf* or valuation* or value? or valuing or view or views or viewpoint* or worrie? or worry*) adj3 (death or health* or life or living)).tw,id. (1016807)

241 ((analys#s or valuation? or value? or valuing) adj3 (conjoint or contingent)).tw,id. (5071)

242 best worst.tw,id. (1126)

243 (choice? adj2 behavio?r*).tw,id. (12986)

244 (choice? adj1 (discrete or experiment*)).tw,id. (10215)

245 (decision* adj3 (analy* or board?)).tw,id. (40373)

246 (decision* adj3 (making or makes or made or undertak*)).tw,id. (502198)

247 (decision* adj3 (aid or aids or support* or tool?)).tw,id. (92091)

248 ((emotion? or feeling?) adj3 thermometer?).tw,id. (361)

249 empower*.tw,id. (93670)

250 (focus group? or interview* or questionnaire? or survey*).tw,id. (4023537)

251 (freedom? or libert*).tw,id. (145728)

252 gambl*.tw,id. (35852)

253 informed choice?.tw,id. (6956)

254 (life adj3 (event? or experience?)).tw,id. (116821)

255 (multi?attribute or multi?criteria).tw,id. (3345)

256 (preference? adj1 (elicit* or scor* or stated)).tw,id. (5448)

257 Prospect Theory/ (433)

258 prospect theor*.tw,id. (1800)

259 ((person$2 or self) adj2 (conceiv* or concept*)).tw,id. (48171)

260 ((person$2 or self) adj2 (determin* or efficac* or help or manag* or support*)).tw,id. (255455)

261 (social* adj1 valu*).tw,id. (9739)

262 exp Communication/ (1316723)

263 ((time$2 or timeliness) adj2 (communica* or info*)).tw,id. (23734)

264 (miscommunicat* or mis-communicat*).tw,id. (3047)

265 (misunderstand* or mis-understand*).tw,id. (20585)

266 (misinform* or mis-inform*).tw,id. (9641)

267 ((influenc* or influential or involv*) adj3 (male? or men or patient or patients or patient-oriented or patient-related or person$2 or personally or user or users or user-oriented or user-related)).tw,id. (301006)

268 Informed Consent/ (151902)

269 (informed adj3 (choice* or choos* or consent* or decid* or decision*)).tw,id. (168977)

270 (consent* adj3 (male? or men or patient or patients or patient-oriented or patient-related or person$2 or personally or user or users or user-oriented or user-related)).tw,id. (49794)

271 ((guide? or guiding or influenc* or influential or make or making or makes or made or shar* or support*) adj2 (choice? or choos* or consent* or decid* or decision*)).tw,id. (638455)

272 (screen* adj3 (choice? or choos* or consent* or decid* or decision*)).tw,id. (10471)

273 or/209-272 [PATIENT PREFERENCES/VALUES/QOL] (11109878)

274 208 and 273 [PROSTATE CANCER - SCREENING - PATIENT PREFERENCES/VALUES/QOL] (22716)

275 (201709* or 201710* or 201711* or 201712* or 2018* or 2019* or 2020* or 2021*).up. (39488421)

276 274 and 275 [UPDATE PERIOD] (11497)

277 276 use medall,emczd (11376)

278 276 not 277 [PSYCINFO RECORDS] (121)

279 98 or 191 or 278 [ALL DATABASES] (4220)

280 remove duplicates from 279 (2827)
