# Supplementary figures and images for: Screening for prostate cancer: protocol for updating multiple systematic reviews to inform a Canadian Task Force on Preventive Health Care guideline update
Source: Syst Rev. 2022 Oct 26;11:230. doi: 10.1186/s13643-022-02099-9 (PMC9609189; doi:10.1186/s13643-022-02099-9)

## Additional file 4: PRESS checklist


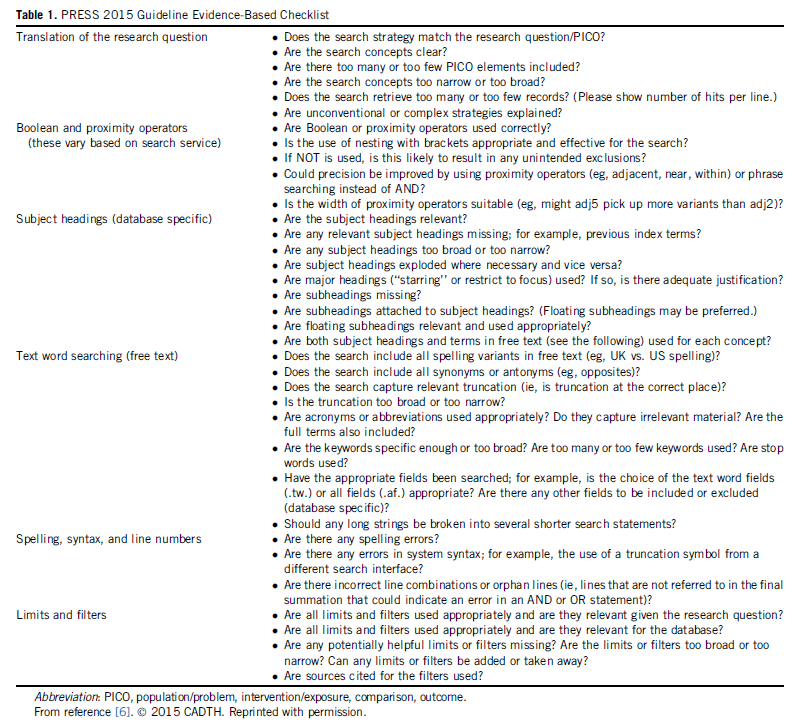

Supplement: Supplementary file 4 — Additional file 4. PRESS checklist. [file 13643_2022_2099_MOESM4_ESM.docx]
