# Supplementary material for: Screening for prostate cancer: protocol for updating multiple systematic reviews to inform a Canadian Task Force on Preventive Health Care guideline update
Source: Syst Rev. 2022 Oct 26;11:230. doi: 10.1186/s13643-022-02099-9 (PMC9609189; doi:10.1186/s13643-022-02099-9)
Supplement: Supplementary file 6 — Additional file 6. Draft data extraction items. [file 13643_2022_2099_MOESM6_ESM.docx]

## Additional file 6: Draft data extraction items

Study characteristics

- authors
- language
- year of publication
- country of study conduct
- number of centres [if applicable]
- study settings and location
- study design
- duration of follow-up
- publication type
- objectives
- funding
- sample size
- sampling technique
- sample size calculation
- study start and end date
- protocol deviations

Population characteristics

- recruitment method
- randomization method
- eligibility criteria for participants
- response rate
- information regarding respondent bias/representativeness of the included population
- baseline demographic and clinical characteristics (e.g., age, sex, gender, socioeconomic status, geographical location, ethnic group, co-morbidities, screening history, family history, immigrant status, definition of elevated PSA test (KQ2))
- number enrolled, assigned to each arm, received intended screening, analyzed, and losses and exclusions after randomization

Details about intervention/exposure

- screening program details (e.g., screening interval, universal/targeted, coverage, setting)
- type of screening test performed, method of collection, positive test threshold
- follow-up tests, biopsy, procedures, care, or treatment
- type of additional testing (KQ2)
- information presented to participants on overall benefits and harms of screening (KQ4)
- information presented in decision aids (KQ4)

Details about comparator

- definition of no screening (KQ1)
- definition of usual care (KQ1, KQ2)
- details on alternate type of screening (KQ1)

Outcomes of interest (see Table 1-3 for KQ1, KQ2, KQ4)

- definition
- measurement methods
- ascertainment methods
- timing

Analysis

- unit of analysis
- statistical methods and additional analyses (adjusted analyses, secondary analyses, missing data)

Results

- number of events
- measures of effect (adjusted and/or unadjusted) and precision (e.g., 95% confidence interval)
- measures of variability
- cluster correlation coefficients (where relevant)
