# Supplementary material for: Screening for prostate cancer: protocol for updating multiple systematic reviews to inform a Canadian Task Force on Preventive Health Care guideline update
Source: Syst Rev. 2022 Oct 26;11:230. doi: 10.1186/s13643-022-02099-9 (PMC9609189; doi:10.1186/s13643-022-02099-9)
Supplement: Supplementary file 7 — Additional file 7. Stakeholder review and feedback. [file 13643_2022_2099_MOESM7_ESM.docx]

## Additional file 7: Stakeholder review and feedback

| **Section** | **Comment** | **Reviewer** | **Response** |
| --- | --- | --- | --- |
| Disease burden and prevalence, pg. 10 | *“The observed decline in mortality may be explained by improved treatment with the introduction of hormonal therapy and advances in radiation therapy.”*  I would say that the cause is truly unknown. In fact, most studies show no change in mortality for metastatic patients and the use of novel systemic therapies are not prevalent enough to cause a change. The same could be said for radiotherapy. Prevalence of EBRT and high dose brachytherapy likely improves cancer survival, but the use is not high enough to be the reason for reduction in mortality. Some of the best work I have seen on this is from Ruth Etzioni’s group which suggest screening (rather than treatment changes) may have a (potentially large) role  (see reference): Tsodikov A, Gulati R, Heijnsdijk EAM, Pinsky PF, Moss SM, Qiu S, de Carvalho TM, Hugosson J, Berg CD, Auvinen A, Andriole GL, Roobol MJ, Crawford ED, Nelen V, Kwiatkowski M, Zappa M, Luján M, Villers A, Feuer EJ, de Koning HJ, Mariotto AB, Etzioni R. Reconciling the Effects of Screening on Prostate Cancer Mortality in the ERSPC and PLCO Trials. Ann Intern Med. 2017 Oct 3;167(7):449-455. doi: 10.7326/M16-2586. Epub 2017 Sep 5. PMID: 28869989; PMCID: PMC5734053.  So, I think a more accurate statement would be, “the cause of a reduction in mortality is unknown, but could be due to decreased incidence or improved screening, treatment, or socioeconomic/environmental factors.” | Reviewer #1 | Thank you for this comment. We have made changes to line 194 to reflect your suggestion. The sentence now reads,  *“The cause of a reduction in mortality is unknown, however could be due to a decreased incidence, improved screening, treatment, or socioeconomic/environmental factors.”* |
| Natural history and risk factors, pg. 10 | *“The disease is heterogenous in its clinical behaviour, with disease progression dependent on tumor stage.”*  For non-metastatic cancer, tumour grade is by far the most informative prognostic factor. PSA level is also independently prognostic. So, I think this should be changed to: The disease is heterogenous in its clinical behaviour, with disease progression dependent on tumor stage, tumour grade, and serum PSA concentration”. | Reviewer #1 | Thank you, we have made the suggested edits. |
| Natural history and risk factors, pg. 10 | The authors have separate sections of incidence/prevalence and natural history/risk factors. But some of the narrative is misplaced (in my opinion). The following paragraph is better in the incidence/prevalence section:  “There are few well-known risk factors for the disease [16]. The incidence of prostate cancer increases with age and rises sharply over the age of fifty [16, 17]. A family history of the disease is associated with increased risk of prostate cancer, with an estimated 20% of cases reporting familial prostate cancer [16, 18]. While this suggests a heritable component, shared environmental risk factors also contribute to this risk [16, 19]. Genome-wide association studies have found several single nucleotide polymorphisms at genetic loci associated with an increased risk of the disease [16, 17, 19]. Individuals with germline mutations in the breast cancer predisposition genes, BRCA2 or BRCA1, have approximately a 20% and 9.5% lifetime risk of developing prostate cancer compared to non-carriers of the mutation, respectively [19]. Individuals may be more susceptible to prostate cancer based on their race and ethnicity. The prevalence of prostate cancer varies widely between racial and ethnic groups, with the highest prevalence among Black Americans with 185 cases per 100,000 compared to 107 cases per 100,000 in White Americans [16–18, 20]. In an American multiple-cohort study, results suggested that Black adults are at an absolute increased risk of prostate cancer–specific mortality of 0.5% (95% CI, 0.2%–0.9%) compared with White men at 10 years post-diagnosis [21]. This  increase in risk may be partially attributed to associated socioeconomic barriers to quality care [16–18, 21].”  I think it is important for the authors to separate risk factors for disease from prognostic factors for patients with disease. Both issues are pertinent to screening. Risk factors for prostate cancer incidence are not well  understood – however, prognostic factors for patients with a diagnosis are well understood with high discrimination and calibration. In my opinion, that does not come out clearly in the introduction. | Reviewer #1 | Thank you, we have considered your suggested edits and adjusted the natural history and risk factors to clarify. |
| Digital rectal examinations, pg. 12 | For the section on DRE – I believe it is important to convey that it is a physical exam test (akin to a breast  exam). As opposed to a PSA (which is objective) DRE is subjective. So, there are 2 considerations – are physicians  competent in performing digital rectal exams? and is DRE useful as a screening test when performed by someone who is competent? As an expert in this field, I am confident that the majority of primary care physicians are not competent in performing DRE. So, even if DRE is useful (I suspect it is), using it as a screening tool is not possible because of inadequate physician training. | Reviewer #1 | Thank you for this comment, we agree and have added your suggested thoughts. The paragraph now reads:  *“The DRE is a physical examination often used alongside the PSA test as a primary screening method in clinical practice [38]. Prior to PSA screening, DRE was the fundamental method of prostate cancer screening, however, by the 1990s, both PSA and DRE were used in conjunction for screening as DRE alone was deemed ineffective to detect prostate cancer [39]. A Canadian survey noted considerable differences in teaching methods for performing DRE, suggesting that using DRE alone as a screening tool is not possible due to inadequate physician training [40].”* |
| Alternative strategies, pg. 13 | In the section for “alternative strategies”, the authors should include PSA density, as this is useful –  especially in contemporary patients with an elevated PSA and screening prostate MRI. | Reviewer #1 | Thank you, we have added a couple sentences outlining the use of PSA density.  *“PSA density, the PSA value (in ng/ml) divided by prostate volume (in CC), is another strategy used to predict prostate cancer, but has not been consistent in daily clinical practice over the years [56]. The use of PSA density has shown to add to the diagnostic value of clinically significant prostate cancer [56, 57].”* |
| PSA tests, pg. 12 | Somewhere in the introduction, it would be worth mentioning the difficulty with PSA screening. Many  studies evaluate a one-point-in-time PSA, where the reality is that for screening/risk assessment, PSA is repeated and considered in conjunction with a number of other factors (symptoms, prostate size). As an example, 25% of patients with an abnormal PSA have a normal PSA when the test is repeated (Lavallée LT, Binette A, Witiuk K, Cnossen S, Mallick R, Fergusson DA, Momoli F, Morash C, Cagiannos I, Breau RH. Reducing the Harm of Prostate Cancer Screening: Repeated Prostate-Specific Antigen Testing. Mayo Clin Proc. 2016 Jan;91(1):17-22. doi: 10.1016/j.mayocp.2015.07.030. Epub 2015 Dec 10. PMID: 26688045.) | Reviewer #1 | Thank you. We have added a section under “PSA tests” to address your comment. The following has been added:  *“Although PSA has been widely used to screen for prostate cancer, there have been some challenges with PSA screening. Many studies have evaluated a one-point-in-time PSA value for screening or risk assessment to make decisions regarding prostate biopsy, however PSA can be repeated and considered in conjunction with other factors such as symptoms or prostate size. One study noted that 25% of patients with an abnormal PSA have a normal PSA when the text is repeated [38].”* |
| Protocol development, pg. 19 | Under protocol development, Andrew Loblaw should not be under University of Ottawa, he must be in  one of the other categories. | Reviewer #1 | Thank you, we have moved Andrew Loblaw to clinical experts on line 452. We have also adjusted the University of Ottawa team to match their respective affiliations. |
| Outcome rating and Table 3, pg. 20-22 | There is an issue of face-validity in outcome rating (in my opinion). I’m a bit puzzled that metastases were  rated as “important” but erectile dysfunction was rated as “critical”. There seems to be a cognitive disconnect here. Clearly, avoiding metastases is critically important to patients, as almost all men with metastases die from prostate cancer and almost all men with prostate cancer are treated with medications that have side-effects. False positives, overdiagnosis and complications due to biopsy are also “critical”, which seems completely out of proportion. “Overdiagnosis” certainly has important implications, but the biggest is “Overtreatment”. Overtreatment is probably a bigger issue here. I would be very surprised if a knowledgeable patient with cancer (or oncologist) to support these designations. How does the committee reconcile “complications due to treatment” as “Important” but erectile dysfunction as “critical”? | Reviewer #1 | Thank you for your comment. For outcome ratings, the Working Group rated a list of outcomes based on GRADE methodology. The calculated means of these votes were organized and categorized as important, critical, or of limited importance. Working Group members may have considered that in trials, metastasis is usually not initially symptomatic, and the outcome of metastases is co-linear with survival. Erectile dysfunction is a unique short-term outcome, and we are discounting outcomes that happen later.  We acknowledge that both erectile dysfunction and metastases are important outcomes, and some can view from a different lens. |
| Table 5, pg. 24 | For KQ2, most of these tests are used to reduce unnecessary biopsy (i.e., false positive PSA) or reduce the  detection of low-grade cancer. However, I do not believe these are listed as outcomes. | Reviewer #1 | Thank you, we have included false positives under harms and will consider these as outcomes. |
| Table 5, pg. 25 | For KQ2 only including the delivery setting of “primary care” may be too restrictive. Most of these  studies are at the intersection of primary care and specialist referral. | Reviewer #1 | Thank you for this point. We have changed our wording to “majority recruited from primary care” as these individuals may be referred to specialists.  We will include trials where the majority are recruited in primary care settings. |
| Table 6, pg. 25 | For KQ3, given informative prognostic factors the population should be stratified based on well established  risk groups. | Reviewer #1 | Thank you. We note that we are using the results of the UK NSC’s review to answer KQ3 and will identify any risk group stratification as outlined by the UK NSC’s systematic review. |
| Table 6, pg. 25 | For KQ3, I believe that watchful waiting or active surveillance should be the comparator (not the  intervention). | Reviewer #1 | Thank you. We note that the UK NSC had monitoring as the intervention for treatment (which included either watchful waiting or active surveillance). After reviewing the UK NSC’s included studies, watchful waiting and active surveillance had been included as a comparator in many trials (e.g., the PIVOT and SPCG-4 trial). Thus, we have included active surveillance and watchful waiting as the comparator. |
| Table 6, pg. 25 | For KQ3, the potential harms should not just include “incontinence”. Patients treated for prostate  cancer can also experience urinary frequency, urinary retention, etc. In addition, erectile dysfunction is not the only sexual complication. For example, nearly 100% of patients treated with androgen deprivation (often used with radiation) experience low libido. | Reviewer #1 | Thank you for your comment. When selecting outcomes, we use the GRADE recommendation for selecting and rating the importance of outcomes. There is a recommended limit on the number of outcomes selected to inform recommendations, however many facets of incontinence may be included. We also note the possibility of capturing some of these outcomes in the outcome of disease specific quality of life, as multiple domains are included in the quality-of-life assessments. |
| Table 6, pg. 25 | For KQ3, why stop the date of publication at 2019? I think the review should be updated, especially  since this is an area that has emerging data. | Reviewer #1 | Thank you for this point. Ideally, we would update KQ3, however, to contain scope and account for limited resources, we have decided not to update. Additionally, we note that no new landmark trials or updates have been published since the publication of the UK NSC’s guidance. There have been advances in the treatment of late-stage disease (metastatic) but not in early disease, and for this KQ, we are focused on early disease (screen detected). |
| General | We know that one of the major areas of controversy in prostate cancer screening trials is control group contamination. This is primarily because north American trials were conducted AFTER screening was already commonly performed. I think it would be appropriate to state this in the protocol. It may be unreasonable for the working group to undertake independent analyses on primary trial data, but they could systematically review work that others have published using trial data (Etzioni and others). | Reviewer #1 | Thank you for noting this. We agree and have added a statement under the risk of bias assessment acknowledging contamination bias. |
| General | I think it is important to acknowledge that while overdiagnosis can lead to overtreatment, this has  become significantly less in the past decade, or more. Numerous studies from around the world (especially Canada and Europe) show that active surveillance is chosen by the majority of contemporary patients who are “overdiagnosed”  (1. Loeb S, Folkvaljon Y, Curnyn C, Robinson D, Bratt O, Stattin P. Uptake of Active Surveillance for Very-Low-Risk Prostate Cancer in Sweden. JAMA Oncol. 2017 Oct 1;3(10):1393-1398. doi: 10.1001/jamaoncol.2016.3600. PMID: 27768168; PMCID: PMC5559339. 2. Cristea O, Lavallée LT, Montroy J, Stokl A, Cnossen S, Mallick R, Fergusson D, Momoli F, Cagiannos I, Morash C, Breau RH. Active surveillance in Canadian men with low-grade prostate cancer. CMAJ. 2016 May 17;188(8):E141-E147. doi: 10.1503/cmaj.150832. Epub 2016 Feb 29. PMID: 26927971; PMCID: PMC4868621). | Reviewer #1 | Thank you for this point. We note that providing active surveillance to mitigate the problems of overdiagnosis is not strong. There is evidence to suggest that most men who choose active surveillance eventually receive active treatment. Active surveillance does result in harms, and it would be interesting to further investigate this.  <https://pubmed.ncbi.nlm.nih.gov/29594024/> |
| Eligibility criteria, pg. 22-27 | I have no specific concerns aside from the fact that there does not seem to be much in terms of specific groups outlined in the PICO table. For example, there  is mention of race/ethnicity, but no attention to paid to geography (potentially differential access to screening and care), or to socioeconomic status (which could also affect access to screening and subsequent treatment, including understanding the complexities of information and trade-offs in undertaking the decision to get prostate cancer screening). | Reviewer #2 | Thank you for your comment. We note that these outcomes are not usually well reported in trials, therefore, to balance scope and account for limited resources, we will evaluate selected characteristics. We will consider these less formally (not during the systematic review) in the evidence to decision making framework. |
| Rationale, key questions, and approach, pg. 17-18 | The research questions are largely focused on the clinical aspects of prostate cancer screening (hence my choice above). However, they do not address the significant question of implementation, cost-effectiveness, and healthcare system sustainability, which are considerations that it seems odd to ignore in this context. | Reviewer #2 | Thank you for your note. We acknowledge the importance of implementation, cost-effectiveness, and healthcare system sustainability. We agree that it would be of interest to provide estimate of costs of care for screening for prostate cancer, however due to the scope of this review and accounting for limited resources, we are unable to conduct a formal cost-effectiveness analysis. Implementation, cost, and sustainability will be informally considered during the evidence to decision framework. |
| Search strategy, pg. 27 | Following from my response to ‘question 3’, if the authors wish to include issues around cost-effectiveness, they may wish to include databases specific to health  economic evaluation, such as that from the UK NHS and University of York (https://www.crd.york.ac.uk/CRDWeb/ShowRecord.asp?ID=32002000739&ID=32002000739 | Reviewer #2 | Thank you for your note, we have included this database in our list of websites to search for grey literature in our Additional file 5. |
| Synthesis of included studies, pg. 34 | My first concern relates to the meta-analysis component of the review. I think the authors should acknowledge that it might not be possible to conduct a meta-analysis  (even in a scenario where there are a sufficient NUMBER of papers). I.e., simply because that threshold of quantity is achieved, there still needs to be some thought given to whether or not a meta-analysis should be conducted. Second, the authors have not included cost-effectiveness studies in their review. This, to me, is an important aspect to the debate around the implementation of any screening program. Some attention is given to preferences and health-related quality of life, which have considerable overlap and an obvious relationship with cost-effectiveness analysis. In my opinion, omitting cost-effectiveness analyses from this review is a major limitation and will significantly limit the opportunity for any potential impact. | Reviewer #2 | Thank you for your comment, we have noted on pg. 34-35 that we will pool study data and perform meta-analyses, if appropriate.  We agree that it would be of interest to provide estimate of costs of care for screening for prostate cancer, however due to the scope of this review and accounting for limited resources, we are unable to conduct a formal cost-effectiveness analysis. Implementation, cost, and sustainability will be informally considered during the evidence to decision framework. |
